# EndHiC: assemble large contigs into chromosome-level scaffolds using the Hi-C links from contig ends

**DOI:** 10.1186/s12859-022-05087-x

**Published:** 2022-12-08

**Authors:** Sen Wang, Hengchao Wang, Fan Jiang, Anqi Wang, Hangwei Liu, Hanbo Zhao, Boyuan Yang, Dong Xu, Yan Zhang, Wei Fan

**Affiliations:** Guangdong Laboratory for Lingnan Modern Agriculture (Shenzhen Branch), Genome Analysis Laboratory of the Ministry of Agriculture and Rural Affairs, Agricultural Genomics Institute at Shenzhen, Chinese Academy of Agricultural Sciences, Shenzhen, 518120 Guangdong China

**Keywords:** Hi-C, Scaffolding, Contig end, Assembly

## Abstract

**Background:**

The application of PacBio HiFi and ultra-long ONT reads have enabled huge progress in the contig-level assembly, but it is still challenging to assemble large contigs into chromosomes with available Hi-C scaffolding tools, which count Hi-C links between contigs using the whole or a large part of contig regions. As the Hi-C links of two adjacent contigs concentrate only at the neighbor ends of the contigs, larger contig size will reduce the power to differentiate adjacent (signal) and non-adjacent (noise) contig linkages, leading to a higher rate of mis-assembly.

**Results:**

We design and develop a novel Hi-C based scaffolding tool EndHiC, which is suitable to assemble large contigs into chromosomal-level scaffolds. The core idea behind EndHiC, which distinguishes it from other Hi-C scaffolding tools, is using Hi-C links only from the most effective regions of contig ends. By this way, the signal neighbor contig linkages and noise non-neighbor contig linkages are separated more clearly. Benefiting from the increased signal to noise ratio, the reciprocal best requirement, as well as the robustness evaluation, EndHiC achieves higher accuracy for scaffolding large contigs compared to existing tools. EndHiC has been successfully applied in the Hi-C scaffolding of simulated data from human, rice and Arabidopsis, and real data from human, great burdock, water spinach, chicory, endive, yacon, and *Ipomoea cairica*, suggesting that EndHiC can be applied to a broad range of plant and animal genomes.

**Conclusions:**

EndHiC is a novel Hi-C scaffolding tool, which is suitable for scaffolding of contig assemblies with contig N50 size near or over 10 Mb and N90 size near or over 1 Mb. EndHiC is efficient both in time and memory, and it is interface-friendly to the users. As more genome projects have been launched and the contig continuity constantly improved, we believe EndHiC has the potential to make a great contribution to the genomics field and liberate the scientists from labor-intensive manual curation works.

**Supplementary Information:**

The online version contains supplementary material available at 10.1186/s12859-022-05087-x.

## Background

The long accurate reads generated by PacBio High-Fidelity (HiFi) technology, with an average read length over 10-Kb and base accuracy over 99%, has been widely applied in the de novo assembly of plant and animal genomes [1]. Specific software for assembling HiFi reads have also been developed, such as Hifiasm [2] and HiCanu [3], which in general can produce large contigs with N50 size over tens of mega-bases for a substantial part of genomes [4–6]. Besides, the ultra-long noisy reads with an average length over 50-Kb from Oxford Nanopore Technology (ONT) and related software development such as wtdbg2 [7], also largely improve the contig continuity in genome assemblies. The huge progress in the contig-level assembly makes the goal of telomere-to-telomere assembly more visible than ever before, however, chromosomal-scale linkage data is still needed to assemble these large contigs into complete chromosomes.

Hi-C sequencing provides a fast and cost-effective way for scaffolding the contigs into chromosome-level sequences [8], based on the facts that the density of Hi-C linked pairs between adjacent contigs is higher than that of between non-adjacent contigs. Several Hi-C scaffolding tools have been developed: LACHESIS and ALLHiC use hierarchical agglomerative clustering algorithm to group contigs, and perform ordering and orientation in the following steps [9, 10]; 3D-DNA cuts contigs in the middle site to form sister-contigs, assigns links to non-sister contigs with reciprocal best requirement, and finishes clustering, ordering and orientation tasks simultaneously [11]; HiC-Hiker aims to improve the contig order and orientation generated by other Hi-C scaffolding tools, adopting a probability model and dynamic programming algorithm [12]; HiRise is specially designed for the in vitro Hi-C data [13], while SALSA2 constructs scaffolds using both edges from the contig graphical fragment assembly (GFA) file and linkages inferred from the Hi-C data [14]. All these tools were mainly tested on the relatively shorter contig assemblies, with contig N50 size equal or lower than 1 Mb, which can be routinely obtained from second-generation accurate short reads or third-generation noisy long reads sequencing. Thanks to the contribution of these pioneer algorithms, hundreds to thousands of chromosomal-scale reference genomes of plants and animals have been successfully generated and published.

Logically, larger contigs will simplify scaffolding with Hi-C data, but it is not as expected for the above Hi-C scaffolding tools. Although they can successfully achieve chromosomal-level scaffolds with the relatively shorter contigs (N50 size ≤ 1 Mb), they will fail to do so with the much larger contigs (N50 size ≥ 10 Mb). A major reason is that all above tools compute the pairwise contig linkages using Hi-C links from the whole contig regions, which is reasonable and suitable for scaffolding of relatively shorter contigs [9–11]. Considering that the Hi-C links of two adjacent contigs concentrate only at the neighbor ends of the contigs (usually < 3-Mb) and large amount Hi-C links also exist between some non-adjacent contigs, the number of Hi-C links between adjacent contigs and that between non-adjacent contigs will become closer when the contig size gets larger. Therefore, the large contig size will reduce the power to differentiate adjacent contig linkages from non-adjacent contig linkages calculated using Hi-C links from the whole contig regions, leading to a high error rate in scaffolding these large contigs. The recently published Pin_hic has realized and tried to resolve this problem [15]. In Pin_hic, each contig is split into 3 equal parts, and only the first and third part are used to represent the 5′- and 3′- ends of the contig, respectively. Beyond Pin_hic, the same research group has been developing another advanced Hi-C scaffold algorithm, YaHS [16], which also adopts the contig end idea to improve the accuracy of the contig contact matrix. Combined with a set of comprehensive graph cleaning methods, it was reported that YaHS can generate genome assemblies with much higher accuracy and contiguity than the previous Hi-C scaffolding tools.

Using the current long accurate HiFi reads, we can routinely generate highly continuous contigs with N50 size approaching or larger than 10 Mb and N90 size approaching or larger than 1 Mb for a certain part of plant and animal genomes [4, 5]. Here we do not include complex genomes, such as polyploidy or those enriched in long repeats, which tend to generate more fragmental contig assemblies. For some simpler genomes with less repeat sequences, the contig assemblies can even achieve near-chromosome level, i.e., most chromosome is just composed of one to five contigs. LACHESIS, ALLHiC and 3D-DNA were not designed and not suitable for scaffolding of these large contigs. Pin_hic and YaHS do not thoroughly implement the contig end idea, and they were also mainly tested on relatively fragmental contig assemblies. Therefore, it is still challenging for scaffolding these large contigs from HiFi reads assembly with all the available Hi-C scaffolding tools. To resolve this problem, here we developed an independent Hi-C scaffolding tool EndHiC, which constructs scaffolds with only Hi-C links from the most effective region of contig ends. To illustrate the EndHiC method, we used the genome data of a medical and vegetable plant great burdock (*Arctium lappa*) sequenced by our group [4], which was EndHiC’s first successful application and also on which the inspiration of using contig end was born.

## Implementation

### Overview

**Fig. 1:**
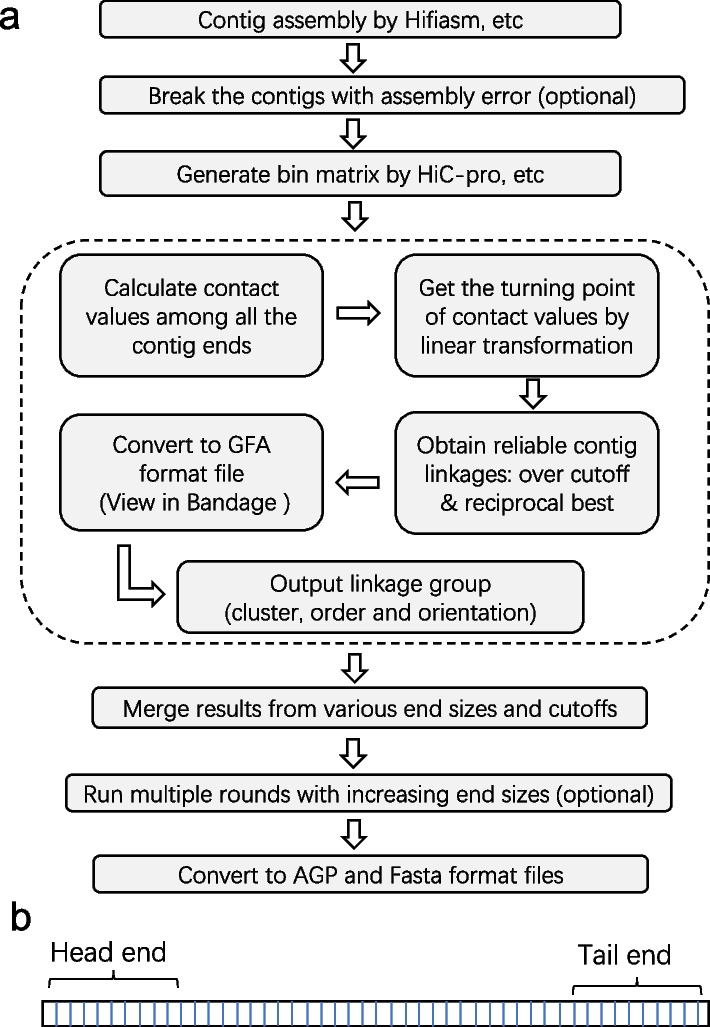
Illustration of EndHiC algorithm; **a** The overall workflow of EndHiC, the five parts inside the dashed lines are the core of EndHiC algorithm, and “optional” means that this step is not necessary for all the cases. **b** Schema chart for contig head and tail end with size 1-Mb, each end contains 10 of 100-kb bins; The last bin of the contig is generally shorter than 100-Kb, but for bin number over 5, i.e. contig end size over 500-Kb, recommended in practice, the size of head and tail ends using specified number of bins can be thought as equal

The overall workflow of EndHiC is shown in Fig. 1a. EndHiC needs two necessary input data: (1) The high continuous contigs generated by Hifiasm [2], HiCanu [3], etc. If the contig correction option is chosen, the contigs will be broken at the mis-joining site when assembly error is detected; (2) The analyzing results of reads mapping from the HiC-Pro pipeline. To fully utilize the professional efforts of HiC-Pro [17], the “.bed” file recording relationships between contigs and bins, the raw and normalized “.matrix” files storing contact values of all bin pairs, with bin size of 100-Kb (recommend), are used by EndHiC.

When the optional module of contig error correction is implemented, EndHiC also needs another optional input data: the mapping file of unitigs to contigs, which can be generated by the EndHiC built-in script “map_utg_to_ctg.pl”. This script reads the HiFiasm output files *_utg.noseq.gfa and *_ctg.noseq.gfa and outputs the mapping relationships of unitigs to contigs. Even without the unitig-to-contig mapping file, EndHiC can still perform contig error correction with only the HiC-Pro output results.

Inside the dashed region of Fig. 1a is the core of EndHiC algorithm, including five steps: (1) The contact value for each pair of contig ends is calculated by summing the contact values of bins falling into the corresponding contig end regions. Each contig has two ends, the left-side end is defined as head end, while the right-side end is defined as tail end. The size of the contig ends is determined as the number of 100-Kb bins (Fig. 1b). (2) Get the turning point of all contact values by a linear transformation method. The turning point is a natural separation of signal and noise contact values. (3) Identify reliable contig linkages which satisfy two requirements: over a specified cutoff and is reciprocal best hits. (4) The contig linkages were converted to GFA format file, which can be graphically displayed in Bandage software [18]. (5) Output the linkage groups (cluster) with order and orientation information.

To increase the accuracy and provide robustness estimation, the scaffolding results with various contig end sizes and contact value cutoffs were merged to generate a consensus scaffolding result, which is generally more reliable. When the contig assembly is quite good, then just one round of EndHiC can produce chromosomal-level scaffolds. However, when the contig assembly is relatively fragmental, multiple rounds of EndHiC with increasing contig end sizes are needed to achieve such goals. Finally, AGP and Fasta format files were generated for downstream applications.

### Eliminate assembly errors in contigs

**Fig. 2:**
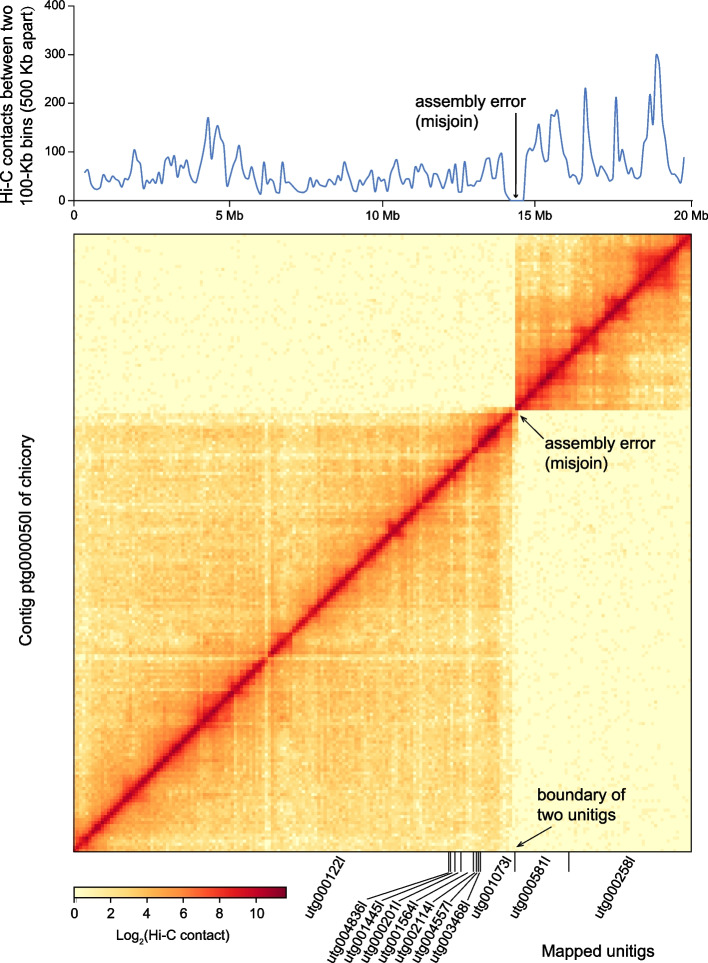
Example of assembly error detection in a contig from chicory. The upper part shows the change of Hi-C contact between two 100-Kb bins 500-Kb apart along the 20-Mb long contig “ptg000050l” from left to right. The lower part is the Hi-C contact heatmap of the contig “ptg000050l”, in which the size of each bin is 100-Kb and the color of each bin is proportional to the Hi-C contact value between two bins. The mapping relationship of unitigs to the contig “ptg000050l” were shown in the horizontal axis. The genomic site with sharp change of Hi-C contact pattern and also is the boundary of two neighbor unitigs, indicates an assembly error

Error-free contigs are important for the scaffold assembly using any Hi-C scaffolding tool. In EndHiC, we detect and correct the contig assembly errors using both Hi-C contact heatmap and the mapping relationship of unitigs to contigs (Fig. 2). Unitigs are the basic non-branching units in the assembly graph constructed from overlapped reads. After complex graph cleaning processes such as tip removal and bubble cleavage, contigs are generated by traversing the linearized paths of unitigs. Therefore, the assembly error in contigs are often caused by incorrect graph cleaning, resulting in mis-join of two non-neighbor unitigs. Previous Hi-C scaffolding tool such as 3D-DNA and YaHS identify the assembly error in contigs based on the intra-contig Hi-C contact heatmap, because mis-join will cause a lack of Hi-C links crossing the point of mis-join. However, we think only using Hi-C is not enough, for that some correctly-assembled genomic regions may also have very low Hi-C links. In EndHiC, we determine the mis-join positions by these two requirements: (1) the Hi-C contact value between two 100-Kb bins (500-Kb apart) is lower than 10% of the median of all 100-Kb bin pairs (500-Kb apart); (2) two separate unitigs surrounding this position. Then, EndHiC will break the contig at the detected mis-join position. When the unitig-to-contig mapping is not available, EndHiC can still perform contig error correction with only the Hi-C contact heatmap, just like 3D-DNA and YaHS do. Note that this step is optional for EndHiC, and you can pass it if you are confident about the accuracy of contig assemblies.

### Estimate the optimal size for contig ends

**Fig. 3:**
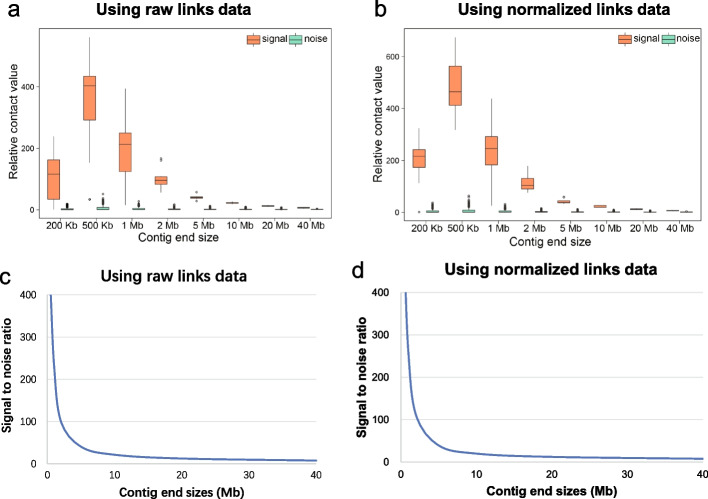
Distribution of signal and noise values using data from great burdock. Boxplot for signal and noise Hi-C contact values along contig end sizes, using the raw links data (**a**) and normalized links data (**b**). For each contig end size, both the signal and noise values are normalized by the median of noise values. Signal to noise ratio (SNR) along contig end sizes calculated from the raw links data (**c**) and normalized links data (**d**). The contact values from adjacent contig ends and non-adjacent contigs ends are taken as signal and noise contact values, respectively. The signal to noise ratio, is defined as the median of signal contact values divided by the median of noise contact values. Both the boxplot distribution and the signal to noise curve showed that the difference between signal and noise contact values will become smaller as the contig end size grows larger

The size of the contig ends is vital for the effectiveness of EndHiC. Then, what is the optimal size value? To answer this question, we calculated the contact values for contig ends with different contig end sizes ranging from 500-Kb to 40-Mb, using the human simulated data and the real great burdock (*Arctium lappa*) genome data that was finely assembled by Hifiasm and further scaffolded into 18 chromosomes [4]. We took the contact values from adjacent contig ends as signal contact values, while that from non-adjacent contigs ends as noise contact values. From the boxplot distribution of the contact values, we observed that the signal and noise contact values were better separated under smaller contig end sizes, but weakly separated under larger contig end sizes (Fig. 3a, b, Additional file 1: Fig. S1a, b).

To quantitatively evaluate the power of using various contig end sizes to differentiate between signal and noise contact values, we further adopted a signal to noise ratio (SNR) approach. For each contig end size, the SNR is defined as the median of signal contact values divided by the median of noise contact values. From the distribution of SNR along contig end sizes, we observed that the SNR is higher than 100: 1 for contig end size less than 2.5-Mb, but becomes lower than 10: 1 for contig end size larger than 20-Mb (Fig. 3c, d, Additional file 1: Fig. S1c, d, Table S1). That is to say, using the contig end sizes larger than 2.5-Mb will have lower ability to differentiate between adjacent and non-adjacent contig linkages. On the other hand, we also noticed that the number of Hi-C links fluctuate severely within smaller contig regions. Thus, the use of contig end sizes smaller than 500-Kb tends to generate instable scaffold results. Therefore, contig end sizes from 500-Kb to 2.5-Mb are preferentially recommended for EndHiC, and the users can change the contig end sizes through parameter settings of EndHiC.

### Determine the turning point of contact values

When the contact values among all the contig ends with a specified size are calculated, the key job is to differentiate the signal from noise contact values. The signal contact values are generated from adjacent contig ends, whereas the noise contact values are generated from non-adjacent contig ends. Is there a cutoff value able to clearly separate the signal and noise contact values? To resolve this question, we sorted all the contact values of contig ends (1.5-Mb) in great burdock, and found a sharp turning point around 1500 in the distribution (Additional file 1: Fig. S2a). The contact values larger than the turning point composes all the signal contact values and a small number of noise signal values, whereas the contact values smaller than the turning point composes only the noise contact values. Based on this finding, we developed a linear transforming method to automatically detect the turning point, which is used as a potential contact cutoff in EndHiC. In the linear transforming method, the adjusted contact values were anticlockwise rotated by 45 degree and the lowest point was identified as the turning point (Additional file 1: Fig. S2bc).

### Scaffolding graph construction and cleaning

**Fig. 4:**
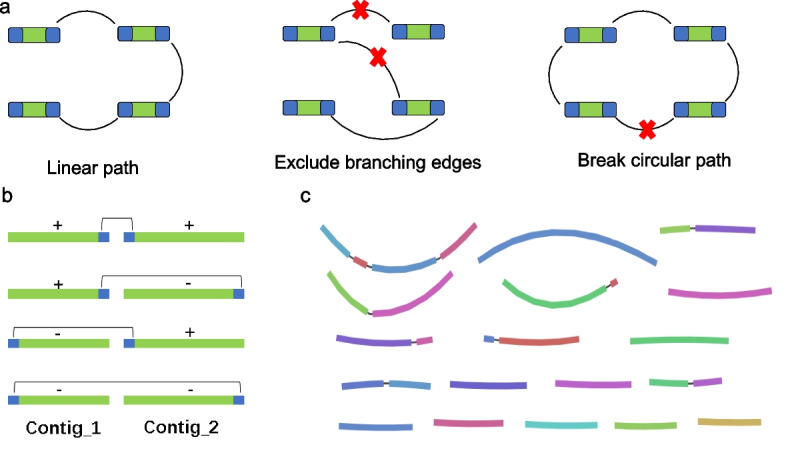
**a** Construction and cleaning of the scaffolding graph. Each contig have two nodes representing the left contig end and the right contig end. The two nodes from the same contig were tied together and won’t be broken, and the two nodes from different contigs if the corresponding contact value is larger than a specified cutoff, were assigned an edge. Branching edges that do not satisfy the reciprocal best requirement will be excluded, and circular paths are broken at the position with lowest contact value. **b** Rules for determining relative contig order and orientation by the reliable links (edge) between neighbor contig ends. The color green and blue indicate contig and contig end, respectively. The connecting line indicate reliable link between adjacent contig ends. The mark “+” and “−” means forward and reverse strands assigned in the GFA file. **c** Example graphic view (Bandage) of cleaned scaffolding graph with contig end size 1.5 Mb and contact cutoff of 1.5 times of the turning point for great burdock, 28 large contigs were linked into 18 linear paths (anchor rate > 99%), with each path corresponding to a natural chromosome. Small clusters with length less than 1 Mb are not shown. Using raw and normalized matrix data generates the same result

WE construct a scaffolding graph with the contig ends as nodes, thus each contig will have two nodes representing the left contig end and the right contig end. The two nodes from the same contig were connected firstly and would not be broken, and then EndHiC assign an edge between two nodes from different contigs if the corresponding contact value is larger than a specified cutoff. As the turning point often falls in the top part of the noise contact values, it is a not a good separation border for the signal and noise contact values. From our experience, we found that 1.5–4.5 times of the automatically identified turning point are more appropriate values to be used as a cutoff here. Lower cutoff tends to cause mis-joining of contigs from different chromosomes, whereas larger cutoff tends to cause segmental assembly of the chromosomes. To clean the graph, EndHiC adopts a reciprocal best approach [11, 14], requiring the contact value between two adjacent contig ends to be the max contact value for both two contig ends. Thus, branching edges that do not satisfy the reciprocal best requirement will be excluded from the scaffolding graph. Given the reciprocal best requirement, one contig end will have only one link to the other contig end, which makes sure that most paths in the scaffolding graph are linear. However, circular path might still occur when the two ends of a chromosome are connected. When this happens, EndHiC will break the circular paths at the position with lowest contact value (Fig. 4a).

Then, EndHiC converts the raw and cleaned scaffolding graphs into separate GFA format files, following the rules showed in Fig. 4b. If the link is assigned to the tail end of contig_1 (on the left side), a strand mark “+” will be given to contig_1, whereas if the link is assigned to the head end of contig_1, a strand mark “−” will be given to contig_1; the opposite rules are applied to contig_2 (on the right side). Conveniently, the GFA file representing the scaffolding graph can be viewed in Bandage, a tool for visualizing assembly graphs with connections [18]. As an example, the Bandage view of cleaned scaffolding graph from great burdock with contig end size 1.5 Mb and contact cutoff of 1.5 times of the turning point is shown in Fig. 4c. With the graphic tool, the users can quickly have an overall view about the scaffolding quality, and find the assembly errors more easily. On this example, the 18 linear paths were displayed, with each path corresponding to a natural chromosome of great burdock. Finally, the resulting cluster file with order and orientation information were generated by traversing each linear path independently on the cleaned scaffolding graph.

### Merge clusters from parallel results with various parameters

To increase scaffolding accuracy, the EndHiC pipeline (endhic.pl) runs in parallel with various contig end sizes from 500 to 2500-Kb in step of 500-Kb, and various contact cutoffs from 1 to 5 times of the basic cutoff (turning point value) in step of 0.5, using both raw and normalized matrix data. Each combination of the parameters generates an independent cluster file with contig order and orientation. Most of these cluster files contain correct chromosomal-scale scaffolds, but a few chromosomes in some cluster files may be mis-scaffolded. Then, EndHiC summarizes all these cluster results (Additional file 1: Table S2), merges the cluster units and counts their frequency. The cluster units were output sequentially based on the frequency from high to low, because cluster units with a relatively higher frequency are thought to be more reliable. During this process, cluster units with contigs that have appeared in previously outputted cluster units were not chosen to output. By this way, a set of non-redundant cluster units is generated as the best result of EndHiC scaffolds. In addition, the frequency of the cluster unit is also used as a robustness evaluation, reflecting the accuracy of each individual cluster unit.

For the great burdock example, the consensus clusters (Table 1) are the same to that shown in Fig. 4c, which just use one parameter combination: contig end size 1.5 Mb and contact cutoff of 1.5 times of the turning point. Although both the consensus method and single parameter method have successfully generated 18 chromosomal-level scaffolds, we strongly recommend the users to run the consensus method which can generate the correct result automatically at most times. In contrast, it will be a little difficult to determine the optimal contig end size and contact cutoff value for a single parameter method, which needs more experience and will not guarantee to generate the correct result every time. When the consensus clusters seem not totally correct, you can also look into the cluster files generated from various parameter combinations and choose the best cluster file from one parameter combination. At last, EndHiC converts the consensus cluster file from all parameter combinations or the best cluster file from one parameter combination into AGP and Fasta format files, which are compatible for most downstream analysis.

**Table 1:** EndHiC consensus clusters for great burdock with robustness information

Cluster_id	Contig count	Cluster length (bp)	Robustness [max:90]	Included contigs with order and orientation
Cluster_A01	1	180,134,246	90	ptg000007l +
Cluster_A02	4	157,781,296	54	ptg000019l + ;ptg000017l + ;ptg000037l + ;ptg000033l-
Cluster_A03	2	137,502,900	81	ptg000003l + ;ptg000004l-
Cluster_A04	2	116,070,885	80	ptg000002l + ;ptg000026l +
Cluster_A05	1	103,325,058	90	ptg000009l +
Cluster_A06	1	98,139,150	90	ptg000005l +
Cluster_A07	2	91,022,879	90	ptg000020l + ;ptg000021l-
Cluster_A08	2	90,391,671	90	ptg000011l-;ptg000023l +
Cluster_A09	2	89,788,315	76	ptg000018l-;ptg000028l +
Cluster_A10	2	83,430,363	89	ptg000010l-;ptg000025l +
Cluster_A11	1	76,614,701	90	ptg000013l +
Cluster_A12	1	76,000,989	90	ptg000012l +
Cluster_A13	1	73,598,797	90	ptg000022l +
Cluster_A14	1	70,868,591	90	ptg000016l +
Cluster_A15	1	69,740,074	90	ptg000001l +
Cluster_A16	2	67,669,029	90	ptg000015l + ;ptg000027l-
Cluster_A17	1	61,985,296	90	ptg000008l +
Cluster_A18	1	60,844,256	90	ptg000014l +

This is the final merged cluster results from one round of EndHiC, with each cluster corresponding to a natural chromosome. Robustness represents the frequency of this cluster type in the EndHiC results of all parameter combinations. For the order and orientation of contigs, “+” stands for the forward strand, while “−” stands for the reverse strand

### Run multiple rounds with increasing contig end sizes

Originally, EndHiC was designed for long continuous contig assemblies, such as the great burdock, on which just one round of EndHiC is able to produce the chromosome-scale scaffolds. The contig end sizes ranging from 500 to 2500-Kb in the default EndHiC parameters are the most effective values. However, even the large contigs might have repetitive sequences on the contig ends, and sometimes the contig assemblies might be a little fragmental than expected. For these cases, one round EndHiC might fail to scaffold all the chromosomes, resulting in non-chromosomal-level scaffolds.

To resolve these problems, we further developed an iterative mode of EndHiC (endhic_iterate.pl), with each iterative round using increased contig end sizes. The following round will use the assembly result of previous round as the input data. By default, the first round still uses contig end sizes from 0.5 to 2.5 Mb, and the following rounds will use contig end sizes 3–5 Mb, 5.5–7.5 Mb, 8–10 Mb, …, with each round increasing 2.5 Mb. Note that the increasing step of the contig end sizes can be set by the users. It should be emphasized that the scaffolding accuracy will decrease as the contig end size grow larger, so the round number no larger than 3, i.e., contig end size no larger than 10 Mb, is recommended in order to get relatively reliable scaffolding results. When the chromosome number is available, you can choose the scaffolding results from the round in which the scaffold number is approaching or equal to the chromosome number. However, if the chromosome number is not known, we do not suggest to run the iterative mode of EndHiC, because it is hard to determine the appropriate round number. EndHiC does not take long scaffold length as its primary goal, but aims to generate a reliable scaffolding result. Furthermore, the EndHiC result files can be converted into Juicebox’s “.assembly” and “.hic” format files, which can be loaded into the graphical viewing tool Juicebox for verification and manual curation [19].

## Results

### Comparison of scaffolding accuracy using simulated and real data

The accuracy of EndHiC was compared with LACHESIS [9], ALLHiC [10], 3D-DNA [11], Pin_hic [15] and YaHS [16] using both the simulated data (human, rice, Arabidopsis) and the real data (human, great burdock, water spinach) (Table 2). In the simulated data, the contigs are simulated from the reference genome (Additional file 1: Table S3), whereas in the real data, the contigs were assembled from HiFi reads. The continuity (N50 size) of the simulated contigs is comparable to those of real contigs that assembled from HiFi reads, in average about 5 contigs per chromosome. In addition, smaller contigs with size less than 1 Mb were excluded. Considering that assembly errors were not added in the simulated contigs and there were no obvious assembly errors in the analyzed contigs of real data, contig error corrections were not performed for all the Hi-C scaffolding tools. All the Hi-C data used here are real sequencing data, with coverage depth ranging from 40 to 130× (Additional file 1: Table S4). The human, rice and Arabidopsis data have been widely used in testing of Hi-C scaffolding tools, while the genomes of two species great burdock and water spinach were recently sequenced by our group.

**Table 2:** Summary of simulated and real contig data

Assembly type	Species	Chromo-somes (n)	Genome size	Total contig number	Total contig length	Assembled percent (%)	Average contig number	Contig N50	Contig N90
Simulated assembly	Human	23	3,054,815,472	104	3,054,815,472	100%	4.52	47,909,438	14,532,355
	Rice	12	373,094,580	42	373,094,580	100%	3.50	11,071,427	5,000,429
	Arabidopsis	5	119,146,348	11	119,146,348	100%	2.20	9,660,775	5,994,203
Hifiasm-assembly	Human	23	3,054,815,472	82	3,007,080,905	98%	3.57	89,131,734	28,203,557
	Great burdock	18	1,720,000,000	30	1,709,056,189	99%	1.67	74,692,580	38,981,084
	Water spinach	15	485,000,000	29	480,197,403	99%	1.61	23,511,778	9,860,712

Genome size are validated or estimated value. The contigs with size > 1 Mb are used for statistics in this table. Assembled percent = total contig length/genome size. Average contig number means average contig number per chromosome. For simulated data, the reference genomes of human CHM13 v1.1, rice (Nipponbare) ASM386523v1, Arabidopsis thaliana (Columbia) TAIR10.1 were used to simulate large contigs, and each chromosome of reference genome was randomly split into 1–6 contigs. In the simulated assembly, contigs size smaller than 1 Mb were not allowed, and all the contigs are larger than 1 Mb. For real data, the hifiasm-assembled contigs of human were downloaded from https://zenodo.org/record/4393631/files/CHM13.HiFi.hifiasm-0.12.fa.gz, while the contigs of great burdock and water spinach were assembled by hifiasm using default parameters from HiFi reads downloaded from NCBI-SRA databases (PRJNA764011 and PRJNA764042)

One round of EndHiC with default parameters is applied to all the datasets except for the human real data, which needs two rounds of EndHiC to achieve chromosome-level scaffolds. In the human real data, some contig ends have relatively lower link density due to long heterochromatin repeat sequences, so the first round (A) of EndHiC with default contig end sizes 0.5–2.5-Mb failed to cluster these contigs into chromosome-level scaffolds, resulting in 34 clusters (Additional file 1: Fig. S3a). Larger contig ends will span the repetitive regions in the contig end and overcome this problem, so the second round (B) using contig end sizes 3.0–5.0-Mb have successfully clustered these contigs, resulting in 23 clusters, which is equal to the chromosome number of human (Additional file 1: Fig. S3b).

We used the corresponding reference genomes to validate the EndHiC assembly accuracy of species human, rice and Arabidposis, and used the Bandage graphic view (Additional file 1: Fig. S4), Hi-C heatmap (Additional file 1: Fig. S5), as well as the synteny relationship with closely related species (Additional file 1: Fig. S6), to validate the EndHiC assembly accuracy of species great burdock and water spinach, whose reference genome are not available. Considering all these evidences, EndHiC have correctly clustered all the simulated and real large contigs into chromosomes. In respect to order and orientation, EndHiC only makes a mistake for two neighbor contigs in chromosome_1 of the human real data, caused by the extremely long heterochromatin repeat sequence (~ 20 Mb) between them. For the anchor rate, EndHiC has anchored 100% of the contigs into chromosomes in simulated data, and anchored 99.3%, 99.8%, 99.7% of the contigs into chromosomes for human, great burdock, and water spinach real data, respectively. These results suggest that EndHiC is able to assemble the large contigs generated from the current HiFi reads into chromosome-level scaffolds, with high accuracy in the cluster, order and orientation, for a certain part of diploid plant and animal genomes.

**Fig. 5:**
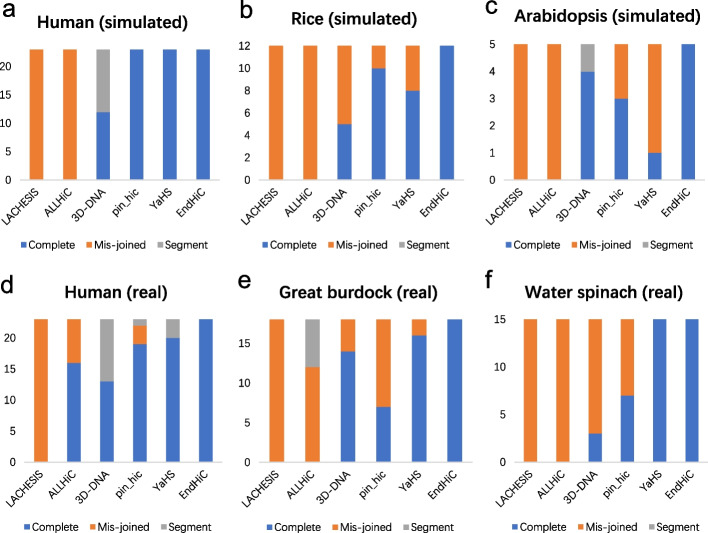
Comparison of clustering accuracy for 6 Hi-C scaffolding tools. LACHESIS and ALLHiC were run with chromosome number as input, and the other Hi-C scaffolding tools do not need the chromosome number. For human (n = 23), rice (n = 12), Arabidopisis (n = 5), the reference genomes of human CHM13 v1.1, rice (Nipponbare) ASM386523v1, Arabidopsis thaliana (Columbia) TAIR10.1 were used for comparison with the results of four Hi-C scaffolders. For great burdock (n = 18) and water spinach (n = 15), the genomes of closely related species *Cynara cardunculus* (NCBI RefSeq GCF_001531365.1) and *Ipomoea triloba* (NCBI RefSeq GCF_003576645.1) were used for comparison. For fairness, only larger contigs with size > 1 Mb were used for all the 6 Hi-C scaffolding tools, and the percent of used contigs were shown in Table 2. Just one round of EndHiC was applied to all the 3 simulated datasets (human, rice, Arabidopsis) and two real datasets (great burdock, water spinach), and two rounds of EndHiC were applied to the human real dataset. Complete: A Hi-C scaffold includes most fragments only from one chromosome, i.e. the total length of shared contigs between a Hi-C scaffold (cluster) and the reference chromosome accounts for over 85% of both the scaffold (cluster) and the chromosome. Segment: A chromosome is fragmented into two or more large scaffold, and each segment scaffold accounts for 5–85% of the corresponding chromosome. Mis-joined: A Hi-C scaffold includes large fragments from two or more chromosomes. In overall, the clustering accuracy for these large contigs is: LACHESIS < ALLHiC < 3D-DNA < Pin_hic < YaHS < EndHiC

In contrast, LACHESIS, ALLHiC, 3D-DNA, Pin_hic and YaHS only correctly clustered 0%, 17%, 53%, 72% and 87% of the chromosomes, respectively, in average of the simulated and real data (Fig. 5, Additional file 1: Table S5). For the round number, there was no iterative scaffolding process for Lachesis and AllHiC, which use hierarchical agglomerative clustering algorithm; Pin_hic iterative model was used, and it iterated three times by default; 3D-DNA and YaHS determines the iterative round number automatically inside the tools. The round number for all the tools on all the datasets were shown in Additional file 1: Table S6. In general, the major problem for LACHESIS and ALLHiC is mis-joining of different chromosomes, whereas the major problems for 3D-DNA, Pin_hic and YaHS are segmental assembly of chromosomes and mis-joining of different chromosomes. Taking the scaffolding results of the human (n = 23) real data as an example, LACHESIS, ALLHiC and Pin_hic mis-joined 23, 7 and 3 chromosomes. In addition, 3D-DNA, Pin_hic and YaHS generated segmental scaffolds for 10, 1 and 3 chromosomes, respectively (Additional file 1: Fig. S7). Similar to EndHiC, none of these tools can assign the two neighbor contigs from chromosome_1 of the human real data into one cluster. For the order and orientation, all these scaffolding tools still work well with the large contigs, and the accuracy is comparable to that of EndHiC. Therefore, the major problem for these tools is insufficient (segment) or excessive (mis-join) clustering, which limits their application ranges. For scaffolding large contigs, in average, the overall performance from worse to better is: LACHESIS < ALLHiC < 3D-DNA < Pin_hic < YaHS < EndHiC.

### Evaluation of the accuracy of contig error detection

To evaluate the accuracy of EndHiC contig error detection, we randomly simulated comparable number of erroneous (with one mis-join error) and error-free (with no error) contigs with comparable contig sizes (> 1 Mb), using the reference genomes of Arabidopsis, rice, and human. The corresponding Hi-C reads were mapped to simulated contigs using HiC-Pro, and EndHiC used the *.bed and *.matrix files to detect mis-join errors since unitigs are not available for simulation. Of the simulated 56, 101, and 221 erroneous contigs for Arabidopsis, rice, and human, EndHiC successfully identified 52, 93, and 200 contigs to have mis-join errors, respectively. Of the simulated 43, 78, and 167 error-free contigs for Arabidopsis, rice, and human, EndHiC predicted 43, 78, and 160 contigs to have no assembly errors, respectively. Overall, the sensitivity and specificity of EndHiC mis-join detection with Hi-C data are both higher than 0.9 (Additional file 1: Tables S7, S8). However, we should be alert that the simulated contig data cannot fully mimic the complexity of genome assembly from real sequencing data. Thus, the accuracy of contig error detection for real data may be different from that of these simulated data.

### Applications of EndHiC in other genome projects

Till now, EndHiC has also been applied to resolve the scaffolding assemblies of several other genomes, including 3 economic plants chicory (*Cichorium intybus*, n = 9), endive (*Cichorium endivia*, n = 9), yacon (*Smallanthus sonchifolius*, n = 29) [4], and an invasive weed *Ipomoea cairica* (n = 15) [5], whose genome sizes are 1.3 Gb, 0.85 Gb, 2.6 Gb, and 0.73 Gb. Applying the contig error correction module of EndHiC, 3, 0, 0, and 1 contigs were identified with assembly errors for chicory, endive, yacon and *Ipomoea cairica*, respectively. These contigs were broken at the assembly error sites before the major Hi-C scaffolding workflow. The contig N50 sizes are 15 Mb, 9 Mb, 87 Mb and 44 Mb, and the contig N90 sizes are 4 Mb, 0.8 Mb, 43 Mb and 23 Mb, for chicory, endive, yacon, and *I.cairica* respectively. It is obvious that the contig continuity for yacon and *I.cairica* is much better that of chicory and endive. Not surprisingly, only one round of EndHiC was able to achieve chromosome-level scaffolds for yacon and *I.cairica* (Additional file 1: Figs. S8, S9), but two rounds of EndHiC can just generate near-chromosome-level scaffolds for chicory and endive, which were further linked to chromosomes by manual works based on the Hi-C heatmaps (Additional file 1: Figs. S10, S11). The final anchoring rate of contigs into chromosomes are 95.8%, 85.6%, 97.7% and 97.9% for chicory, endive, yacon, and *I.cairica* respectively (Additional file 1: Table S9), most of which are comparable or higher than those of previously published genomes, suggesting that EndHiC can be applied to a broad range of common diploid genomes.

Through in-depth analysis, we found that the chicory and endive genomes contain higher amount of long tandem repeats than yacon and *I.cairica* [4, 5], which largely explains the differences in contig continuities. The existing of long tandem repeats in some contig ends, as well as the relatively larger gap sizes between neighbor contigs for chicory and endive, may be the major reasons why EndHiC works less well on chicory and endive. Otherwise, the excellent performance on yacon and *I.cairica* showed again that EndHiC is suitable for scaffolding of very large and even up to near-chromosome contigs. In these genome projects, the authors have also tried to use the other available Hi-C scaffolding tools and found that they all can’t generate chromosome-level scaffolds automatically without manual curation, suggesting that EndHiC is more suitable than previous Hi-C scaffolding tools on the applications of these large contigs with N50 size near or over 10 M and N90 size near or over 1 Mb.

### Evaluation of EndHiC performance on contig assemblies with various length

TO further investigate the scope of application for EndHiC, we simulated human contigs with various length and analyzed the scaffolding results from EndHiC (Additional file 1: Table S10). These results clearly showed that when the contigs are very large with contig N50 size over 40 Mb, i.e., the average contig number per chromosome less than 5, just one round of EndHiC is able to achieve chromosome-level assembly. For moderate large contigs with contig N50 size between 20 and 40 Mb, i.e., the average contig number per chromosome between 5 and 10, two to three rounds of EndHiC can achieve chromosome-level assembly. For lower large contigs with contig N50 size between 10 and 20 Mb, i.e., the average contig number per chromosome between 10 and 30, three rounds of EndHiC can successfully scaffold most of the human chromosomes. However, for relatively shorter contigs, with contig N50 size less than 10 Mb, EndHiC can’t get chromosome-level or near-chromosome-level assembly automatically. In addition, the assembly error may increase as the contigs become shorter. Therefore, we suggest that EndHiC should only be applied to contig assemblies with an N50 size near or over 10 Mb and an N90 size near or over 1 Mb. For assemblies with relatively shorter contigs, other Hi-C scaffolding tools such as 3D-DNA, Pin_hic, and YaHS have been proved to be more suitable and can generate better assembly results [11, 15, 16].

### Application of EndHiC to ONT-assembled contigs

To evaluate the performance of EndHiC on the ONT data, an ONT-assembled human (CHM13) genome by Shasta was used [2, 20]. The total contig length of this ONT-assembly is 2.72 Gb, which is ~ 10% shorter than the human reference genome (3 Gb), indicating that about 300 Mb sequences have been missed from the contig assembly. After filtering the smaller contigs with size less than 1 Mb, we obtained a total contig size of 2.64 Gb, including 130 contigs with a N50 size of 48.8 Mb and a N90 size of 10.7 Mb, which was used for testing the performance of EndHiC. From the Hi-C heatmaps of contigs, we observed no obvious assembly errors in the contigs (Additional file 1: Fig. S12a), so the contig error correction module of EndHiC was not applied. Then, three rounds of EndHiC with default parameters were applied to acquire the 23 clusters, whose number is equal to the chromosome number of human (CHM13), and the three rounds of EndHiC resulted in a total of 44, 26, and 23 clusters, respectively (Additional file 1: Fig. S12b, c). In the final 23 clusters, up to 98.6% of the used contig sequences have been anchored to the chromosomes.

Most of the contigs were in correct order and orientation except that in Chr1, Chr9 and Chr17 of human (CHM13), mainly caused by missing large fragment between the neighbor contigs (Chr1 miss 34.4 Mb, Chr9 miss 42.6 Mb, Chr17 miss 26.8 Mb) (Additional file 1: Fig. S12d). Compared to the EndHiC results of HiFi-assmebled human genome, one more round of EndHiC was performed here, and there are two more chromosomes with contig order and orientation problem in the ONT-assembled human genome, suggesting that EndHiC is applicable to the scaffolding of ONT-assembled contigs but more assembly errors may occur compared to the scaffolding of HiFi-assembled contigs. However, along with the continuous improvement of the ONT contig assembly, we believe that EndHiC will generate better scaffolding results with the ONT-assembled contigs in future.

### Performance comparison in memory and time

Although written in Perl, EndHiC has very high efficiency in the computer memory and CPU time due to innovative algorithm. Generally, only a few minutes and less than 1-G memory is needed by EndHiC for most genomes. The speed of EndHiC is ~ 10 times faster than LACHESIS, ALLHiC, Pin_hic and YaHS, and ~ 1000 times faster than 3D-DNA. In addition, the memory consumption of EndHiC is also the lowest among all the Hi-C scaffolding tools (Table 3). We did not include the preprocessing steps (reads mapping and filtering) here for all the compared tools, so it is fair for the comparison of the core Hi-C scaffolding algorithm among each tool. Generally, the preprocessing steps consumed much more time (10–10,000 times) than the core Hi-C scaffolding algorithm, and HiC-pro takes slightly more time (1–6 times) than the other preprocessing methods (Additional file 1: Table S11). Moreover, EndHiC is easy to setup and use, with no dependent packages and fewer parameters needed to be determined by the users at most cases, and the human-readable text-format cluster file as well as the graphic GFA format output file bring great convenience to the users.

**Table 3:** Performance comparison for EndHiC with other 5 Hi-C scaffolding tools

Hi-C scaffolder	Human			Rice			Arabidopsis		
	Elapsed time (m)	CPU time (m)	Peak memory (Gb)	Elapsed time (m)	CPU time (m)	Peak memory (Gb)	Elapsed time (m)	CPU time (m)	Peak memory (Gb)
LACHESIS	5.6	5.6	2.7	9.9	9.9	0.4	1.5	1.5	0.30
ALLHiC	22.3	43.2	38.2	17.1	40.7	16.8	3.2	7.0	2.80
3D-DNA	1,314.0	2,100.0	51.6	354.0	816.0	51.6	102.0	534.0	28.70
Pin_hic	171.7	150.3	5.6	24.6	22.9	0.6	15.8	14.2	0.60
YaHS	29.7	29.4	7.0	5.0	3.7	0.4	1.6	1.6	0.14
EndHiC	0.5	3.6	0.2	0.2	1.1	0.1	0.1	0.6	0.02

All the statistics here do not include the preprocessing steps (reads mapping and filtering), so it is fair to compare the major Hi-C scaffolding algorithms among the 6 tools. The simulated contig data of three model species shown in Table 2 were used to test EndHiC, LACHESIS, ALLHiC, 3D-DNA, Pin_hic and YaHS. For all these simulated datasets, just one round of EndHiC was applied. Note that LACHESIS only finished the cluster step and then exited, it did not perform order and orientation here. The reason might be that LACHESIS grouped all the simulated large contigs into a single cluster and produced invalid intermediate files for later steps. Based on previous experience, the speed and memory consumption of LACHESIS is comparable to that of ALLHiC

## Discussion

EndHiC implements a simple but effective Hi-C scaffolding algorithm specially for large contigs, leveraging on the HiC-Pro professional works for valid pair detection, calculation and normalization of bin matrix [17]. Unlike LACHESIS [9] and ALLHiC [10], the core algorithm of EndHiC is more similar to 3D-DNA [11], Pin_hic [15] and YaHS [16], which all break the contigs into parts and perform clustering, ordering, and orientation simultaneously. Besides, none of them needs the chromosome number as input. By using only the Hi-C links from the most effective region of the contig ends, EndHiC has a greater SNR compared to other available Hi-C scaffolding tools, contributing mostly to the higher scaffolding accuracy. The scaffolding results from using various contig end sizes and various contact cutoffs with both raw and normalized links data, have also been compared and merged to get a more accurate consensus assembly as well as a robustness evaluation. For genomes with a high proportion of heterochromatin, long repetitive sequences often exist in the contig ends, or genomes with relatively fragmental contig assemblies, one round of EndHiC may fail to assemble all the large contigs into chromosome-level scaffolds. Under these circumstances, we suggest iteratively running multiple rounds of EndHiC, each round with increasing contig end sizes, until the resulting cluster number is equal or close to the known chromosome number. Similarly, 3D-DNA, Pin_hic and YaHS also have the multiple iteration mode.

Unlike the previously developed Hi-C scaffolding tools that are mostly suitable for scaffolding relatively shorter contigs with contig N50 size near or less than 1 Mb, EndHiC is specially designed and has great advantage for scaffolding of much larger contigs. For a certain part of the diploid plant and animal genomes, the contig assemblies derived from the current HiFi sequencing technologies generally reach an N50 size over 10 Mb and an N90 size over 1 Mb. As EndHiC only process contigs with size larger than two times of the contig end size (2 * 500 Kb = 1 Mb), in this case, the majority of the contig sequences (> 90%) are analyzed by EndHiC. Thus, the N90 size requirement is more important than N50 size requirement. To ensure the success of EndHiC, the contig N90 size should be approaching or larger than 1 Mb. The excluded smaller contigs with size lower than 1 Mb are likely rich in repeat sequences and have low Hi-C link density, therefore, by removing the interference of small repetitive contigs that may cause mis-assemblies, it also assures the assembly accuracy of those larger contigs, which forms a correct chromosome framework. Then, the EndHiC package provides a mapping program to assign these small contigs into each of the chromosome frameworks based on the overall maximum Hi-C links. Compared to large contigs, the order and orientation for these small repeat-rich contigs is more difficult to determine, and our contig end rule no longer works. In future, we will try to improve this by further investigations of the small contig data and developing more powerful algorithms.

The chromosome-scale genome assemblies, especially telomere-to-telomere assembly, has been the primary goal for most genome projects. Thanks to the HiFi long-accurate read sequencing technology and the Hifiasm assembling algorithm, we got a surprisingly good contig assembly in the great burdock genome project, with a contig N50 size of 75 Mb and a contig N90 size of 39 Mb. Notably, the total length of contigs with size larger than 1 Mb occupy over 99% of the whole contig assemblies. However, depression follows excitement. With such large contigs, we are still unable to assemble them into correct chromosome-level scaffolds with all available Hi-C scaffolding tools. Then, we found that all these software have used the entire region or a large part of the contig region to calculate Hi-C links among contigs, and suspected that this may be the problem why they failed on scaffolding of these large contigs. To validate our suspicion, we developed the EndHiC algorithm and successfully resolved the chromosome-level assembly of the great burdock. After that, we applied EndHiC to a set of genomes sequenced by our group and our partners, including chicory, endive, yacon, *Ipomoea cairica*, etc., and all achieved chromosome-level or near-chromosome-level scaffold assemblies [4, 5]. During these practices, the default parameters of EndHiC were obtained and optimized, which may be applied to a broad range of genomes.

Although someone may think that scaffolding of such large or even near-chromosome contigs is a trivial work, which can be done directly by manual curation with the Hi-C heatmaps in a graphical tool such as Juicebox [19], we insisted that automatic inferring of the chromosome-level scaffolds like EndHiC is important. As the sequenced genomes become more and more, automatic inferring will save a lot of labor and time. In addition, automatic inferring will also be more reliable than the human eyes. It is worth emphasizing that EndHiC is not going to replace previous Hi-C scaffolding tools, but can be complemented with them. EndHiC is only suitable for contig assemblies with N50 size near or larger than 10 Mb and N90 size near or larger than 1 Mb. With EndHiC, only contigs with size larger than 1 Mb will be assembled into the chromosome framework. Then, you can use EndHiC’s build-in program to assign the leaving small contigs back to each chromosome by the maximum Hi-C links rule, but for the ordering and orientating of the leaving small contigs, currently we suggest apply the global optimization algorithm implemented in HiC-Hiker [12] or the genetic algorithm implemented in AllHiC [10], to obtain the acceptable order and orientation for these small contigs. In contrast, when the contig N50 size is relatively small (< 10 Mb), previous developed tools 3D-DNA, Pin_hic, YaHS, etc. can be adopted firstly. If they failed to generate chromosome-level assemblies, then the resulting large scaffolds can be fed to EndHiC to try to get the chromosome-level scaffolds.

## Conclusion

We developed an independent scaffolding tool EndHiC, which is suitable for scaffolding of long-continuous contig assemblies with a contig N50 size near or over 10 Mb and a contig N90 size near or over 1 Mb. The core idea behind EndHiC, which distinguishes it from other Hi-C scaffolding tools, is using Hi-C links only from the most effective regions of contig ends. By this way, the signal and noise contact values from the corresponding neighbor and non-neighbor contigs are separated more clearly, which makes it easier for the scaffolding graph cleaning and improves the scaffolding accuracy. For performance, EndHiC runs extremely fast with trivial memory consumption, and is easy to use. In summary, the reliable cutoff of contact values, the reciprocal best requirement, the GFA graphic viewing in Bandage, the robustness evaluation, and the compatibility with Juicebox for manual curation, ensure the high scaffolding accuracy of EndHiC. As more genome projects have been launched and the contig continuity constantly improved, we believe that EndHiC has the potential to make a great contribution to the genomics field and liberate the scientists from labor-intensive manual curation works.

## Availability and requirements

Project name: EndHiC.

Project home page: https://github.com/fanagislab/EndHiC.

Operating system(s): Linux, MacOS.

Programming language: Perl, Python

Other requirements: HiC-pro.

License: GNU GPL.

Any restrictions to use by non-academics: None.

## Supplementary Information

**Additional file 1. **Supplementary figures and tables.

## Data Availability

For simulated data, the reference genomes of human CHM13 v1.1 (https://github.com/marbl/CHM13), rice (Nipponbare) ASM386523v1 (https://www.ncbi.nlm.nih.gov/assembly/GCA_003865235.1/), Arabidopsis thaliana (Columbia) TAIR10.1 (https://www.ncbi.nlm.nih.gov/assembly/GCF_000001735.4/) were used to simulate large contigs. For real data, the hifiasm-assembled contigs of human (CHM13) were downloaded from https://zenodo.org/record/4393631/files/CHM13.HiFi.hifiasm-0.12.fa.gz, while the contigs of great burdock and water spinach were assembled by hifiasm using default parameters from HiFi reads downloaded from NCBI-SRA databases (PRJNA764011 and PRJNA764042). The ONT-assembled contigs of human (CHM13) were downloaded from https://zenodo.org/record/4393631/files/CHM13.ONT.Shasta-0.1.0_Helen.fa.gz. The testing data of EndHiC is also available at: https://github.com/fanagislab/EndHiC/tree/master/z.testing_data.

## Abbreviations

HiFi: High-fidelity
ONY: Oxford nanopore technology
GFA: Graphical fragment assembly
SNR: Signal to noise ratio

## References

[1] Marx V (2021). Long road to long-read assembly. Nat Methods.

[2] Cheng H, Concepcion GT, Feng X, Zhang H, Li H (2021). Haplotype-resolved de novo assembly using phased assembly graphs with hifiasm. Nat Methods.

[3] Nurk S, Walenz BP, Rhie A, Vollger MR, Logsdon GA, Grothe R, Miga KH, Eichler EE, Phillippy AM, Koren S (2020). HiCanu: accurate assembly of segmental duplications, satellites, and allelic variants from high-fidelity long reads. Genome Res.

[4] Fan W, Wang S, Wang H, Wang A, Jiang F, Liu H, Zhao H, Xu D, Zhang Y (2022). The genomes of chicory, endive, great burdock and yacon provide insights into Asteraceae palaeo-polyploidization history and plant inulin production. Mol Ecol Resour.

[5] Jiang F, Wang S, Wang H, Wang A, Xu D, Liu H, Yang B, Yuan L, Lei L, Chen R (2022). A chromosome-level reference genome of a Convolvulaceae species *Ipomoea cairica*. G3 (Bethesda).

[6] Wang S, Wang A, Wang H, Jiang F, Xu D, Fan W. Chromosome-level genome of a leaf vegetable *Glebionis coronaria* provides insights into the biosynthesis of monoterpenoids contributing to its special aroma. DNA Res. 2022. 10.1093/dnares/dsac036 PMC9724771 36197084

[7] Ruan J, Li H (2020). Fast and accurate long-read assembly with wtdbg2. Nat Methods.

[8] Lieberman-Aiden E, van Berkum NL, Williams L, Imakaev M, Ragoczy T, Telling A, Amit I, Lajoie BR, Sabo PJ, Dorschner MO (2009). Comprehensive mapping of long-range interactions reveals folding principles of the human genome. Science.

[9] Burton JN, Adey A, Patwardhan RP, Qiu R, Kitzman JO, Shendure J (2013). Chromosome-scale scaffolding of de novo genome assemblies based on chromatin interactions. Nat Biotechnol.

[10] Zhang X, Zhang S, Zhao Q, Ming R, Tang H (2019). Assembly of allele-aware, chromosomal-scale autopolyploid genomes based on Hi-C data. Nat Plants.

[11] Dudchenko O, Batra SS, Omer AD, Nyquist SK, Hoeger M, Durand NC, Shamim MS, Machol I, Lander ES, Aiden AP (2017). De novo assembly of the Aedes aegypti genome using Hi-C yields chromosome-length scaffolds. Science.

[12] Nakabayashi R, Morishita S (2020). HiC-Hiker: a probabilistic model to determine contig orientation in chromosome-length scaffolds with Hi-C. Bioinformatics.

[13] Putnam NH, O'Connell BL, Stites JC, Rice BJ, Blanchette M, Calef R, Troll CJ, Fields A, Hartley PD, Sugnet CW (2016). Chromosome-scale shotgun assembly using an in vitro method for long-range linkage. Genome Res.

[14] Ghurye J, Rhie A, Walenz BP, Schmitt A, Selvaraj S, Pop M, Phillippy AM, Koren S (2019). Integrating Hi-C links with assembly graphs for chromosome-scale assembly. PLoS Comput Biol.

[15] Guan D, McCarthy SA, Ning Z, Wang G, Wang Y, Durbin R (2021). Efficient iterative Hi-C scaffolder based on N-best neighbors. BMC Bioinform.

[16] Zhou C, McCarthy SA, Durbin R. YaHS: yet another Hi-C scaffolding tool. bioRxiv. 2022. 10.1093/bioinformatics/btac808 PMC9848053 36525368

[17] Servant N, Varoquaux N, Lajoie BR, Viara E, Chen CJ, Vert JP, Heard E, Dekker J, Barillot E (2015). HiC-Pro: an optimized and flexible pipeline for Hi-C data processing. Genome Biol.

[18] Wick RR, Schultz MB, Zobel J, Holt KE (2015). Bandage: interactive visualization of de novo genome assemblies. Bioinformatics.

[19] Durand NC, Robinson JT, Shamim MS, Machol I, Mesirov JP, Lander ES, Aiden EL (2016). Juicebox provides a visualization system for Hi-C contact maps with unlimited zoom. Cell Syst.

[20] Shafin K, Pesout T, Lorig-Roach R, Haukness M, Olsen HE, Bosworth C, Armstrong J, Tigyi K, Maurer N, Koren S (2020). Nanopore sequencing and the Shasta toolkit enable efficient de novo assembly of eleven human genomes. Nat Biotechnol.

