# Supplementary material for: EndHiC: assemble large contigs into chromosome-level scaffolds using the Hi-C links from contig ends
Source: BMC Bioinformatics. 2022 Dec 8;23:528. doi: 10.1186/s12859-022-05087-x (PMC9730666; doi:10.1186/s12859-022-05087-x)
Supplement: Supplementary file 1 — Additional file 1. Supplementary figures and tables. [file 12859_2022_5087_MOESM1_ESM.docx]

## Supporting Information for

**EndHiC: assemble large contigs into chromosome-level scaffolds using the Hi-C links from contig ends**

Sen Wang^†^, Hengchao Wang^†^, Fan Jiang^†^, Anqi Wang, Hangwei Liu, Hanbo Zhao, Boyuan Yang, Dong Xu, Yan Zhang^*^, Wei Fan^*^

Guangdong Laboratory for Lingnan Modern Agriculture (Shenzhen Branch), Genome Analysis Laboratory of the Ministry of Agriculture and Rural Affairs, Agricultural Genomics Institute at Shenzhen, Chinese Academy of Agricultural Sciences, Shenzhen, Guangdong, 518120, China.

*These authors contributed equally to this work. Correspondence should be addressed to [fanwei@caas.cn](mailto:fanwei@caas.cn) or milrazhang@163.com.


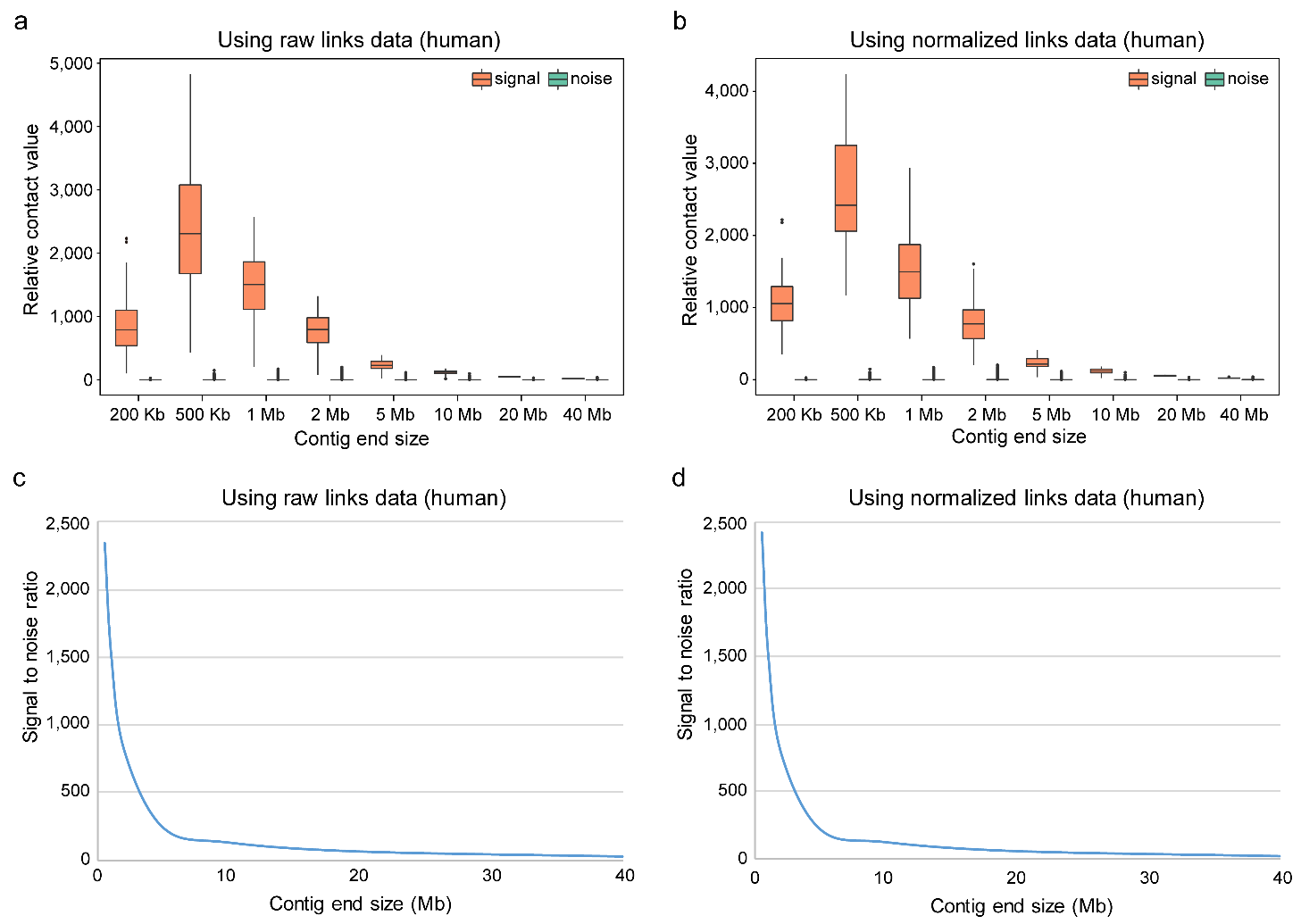


**Figure S1**. Distribution of signal and noise values using data from human simulated data (statistics shown in Table 2). Boxplot for signal and noise Hi-C contact values along contig end sizes, using the raw links data (a) and normalized links data (b). For each contig end size, both the signal and noise values are normalized by the median of noise values. Signal to noise ratio (SNR) along contig end sizes calculated from the raw links data (c) and normalized links data (d). The contact values from adjacent contig ends and non-adjacent contigs ends are taken as signal and noise contact values, respectively. The signal to noise ratio, is defined as the median of signal contact values divided by the median of noise contact values. Both the boxplot distribution and the signal to noise curve showed that the difference between signal and noise contact values will become smaller as the contig end size grows larger, which is consistent with the analysis results from the great burdock shown in Figure 3.


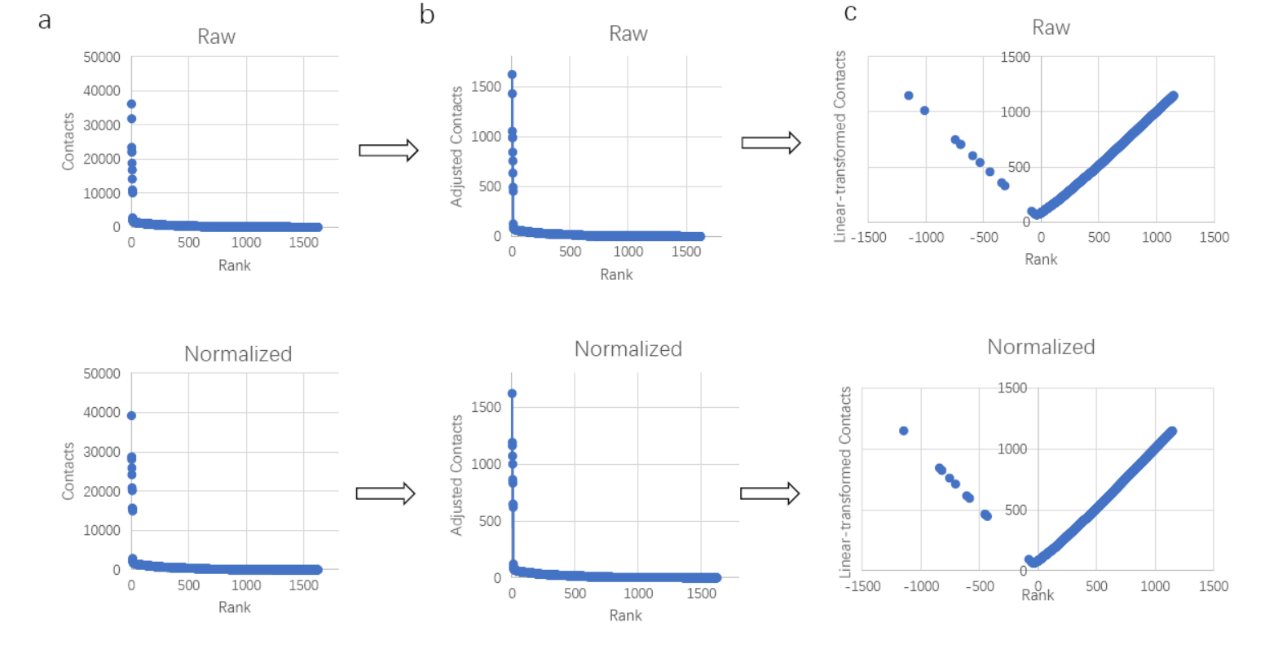


**Figure S2.** Automatic detection of the turning point in sorted contact values of contig ends. Top part uses raw links from HiC-Pro, bottom part uses normalized links from HiC-Pro. a. Distribution of sorted contact values, from max to min. b. Distribution of adjusted contact values, the scale of Y-axis is adjusted to be equal to that of X-axis. c. Distribution of linear-transformed (anticlockwise rotate 45 degree) contact values. The lowest point in the transformed data is much easier to be identified, which is equivalent to the turning point in the original data.


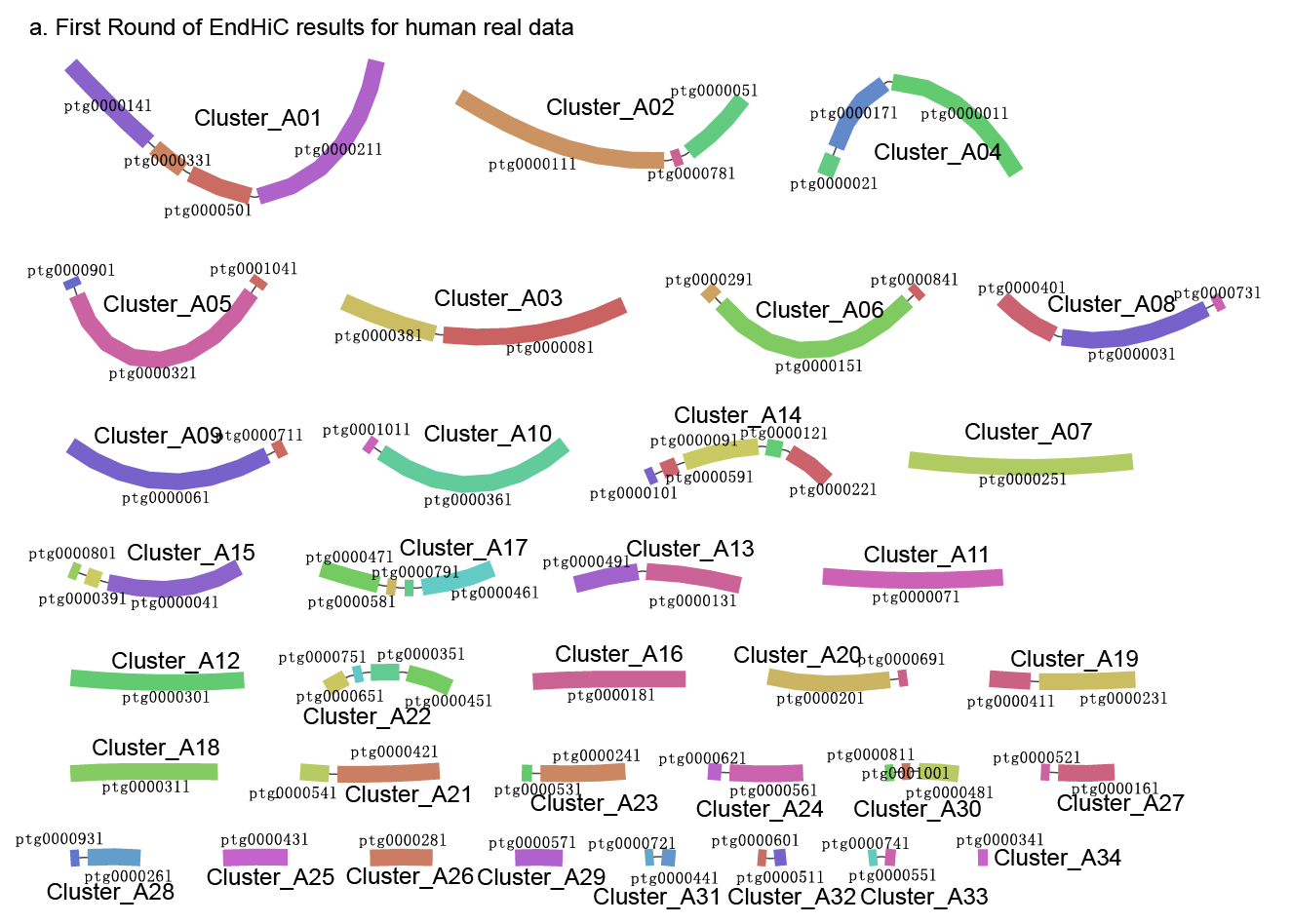


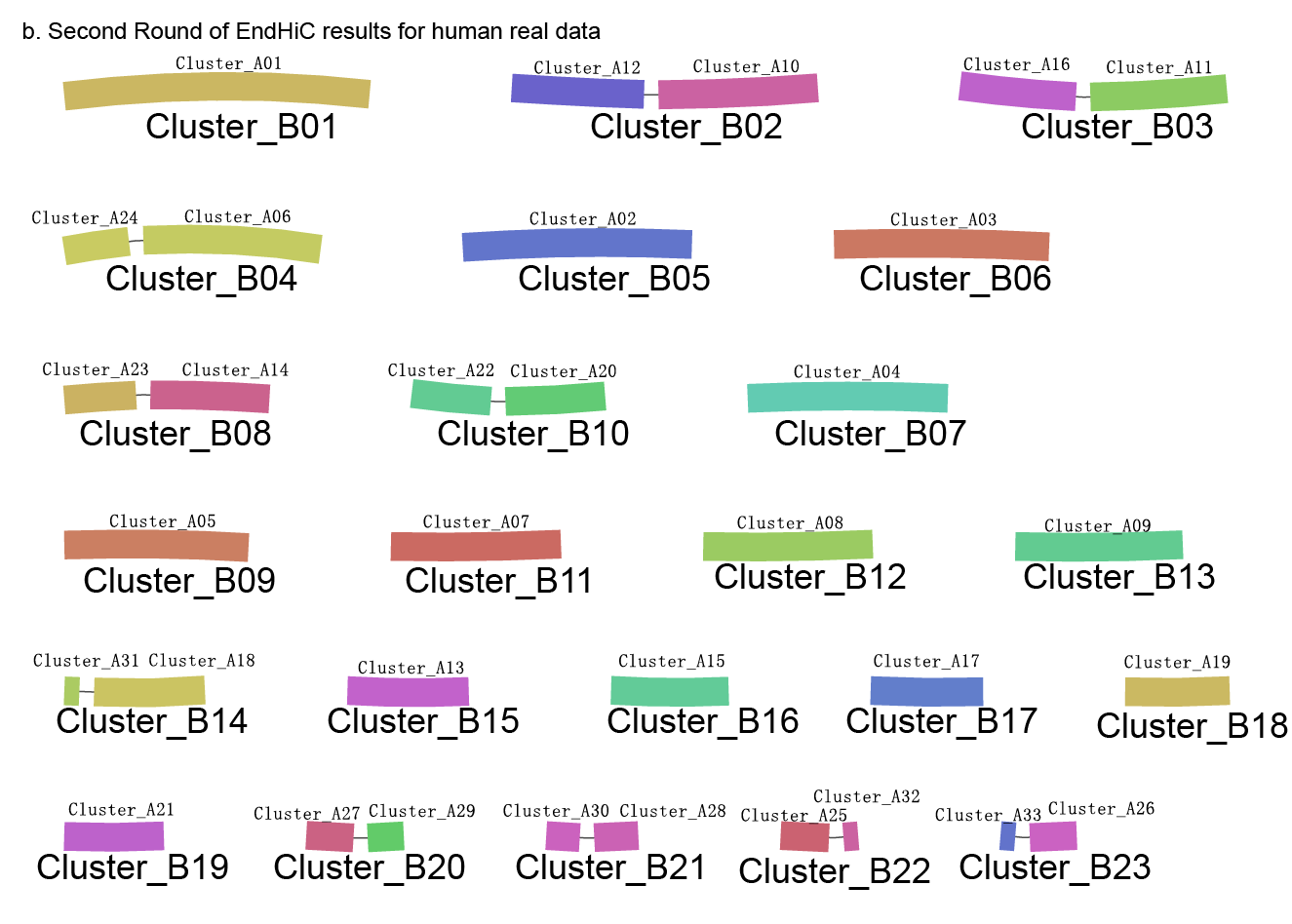


**Figure S3**. Bandage view of EndHiC results for human real data. a. The first round of EndHiC results using contig end sizes 0.5-Mb to 2.5-Mb, all the contigs are assembled into 34 scaffolds (prefix Cluster_A). b. The second round of EndHiC results using contig end sizes 3.0-Mb to 5.0-Mb, all the scaffolds from the first round are assembled into 23 scaffolds (prefix Cluster_B).


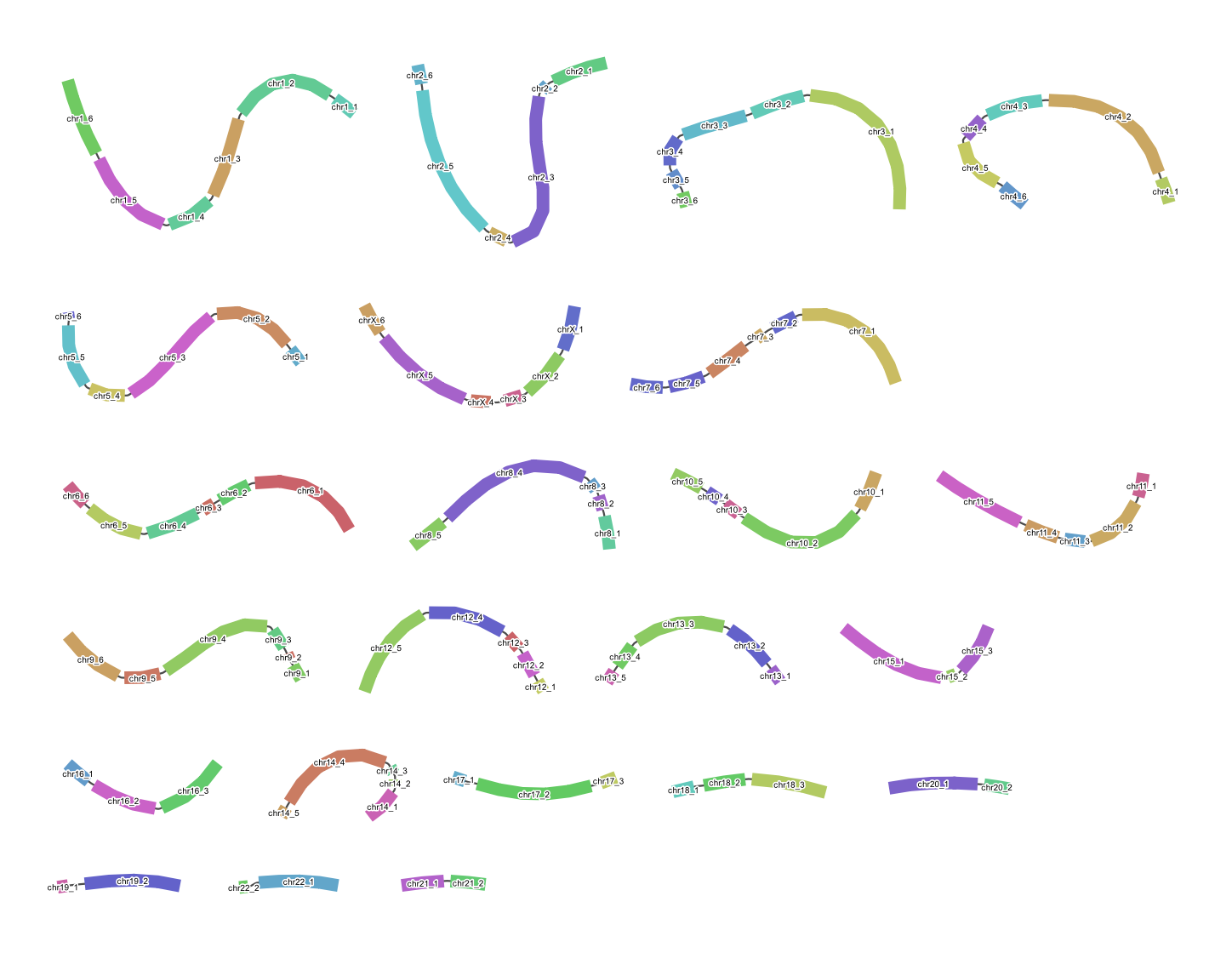


Human simulated data: EndHiC cluster


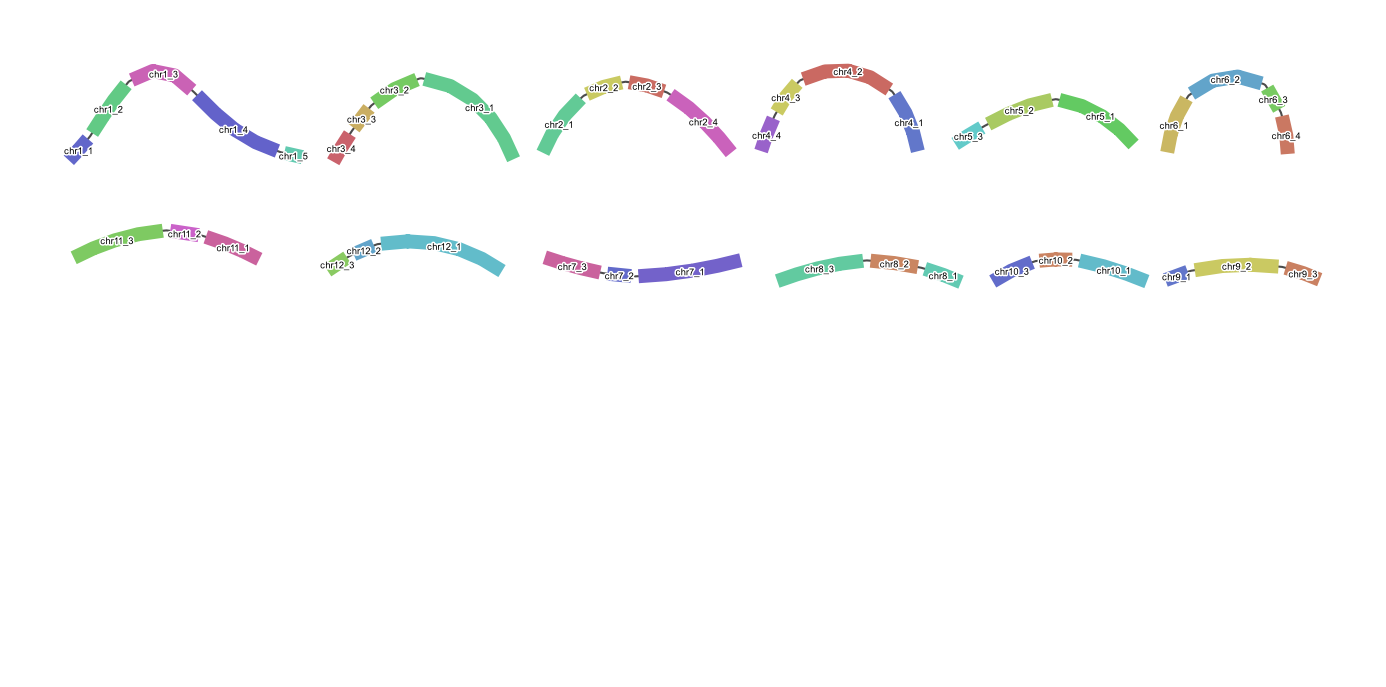


Rice simulated data: EndHiC cluster


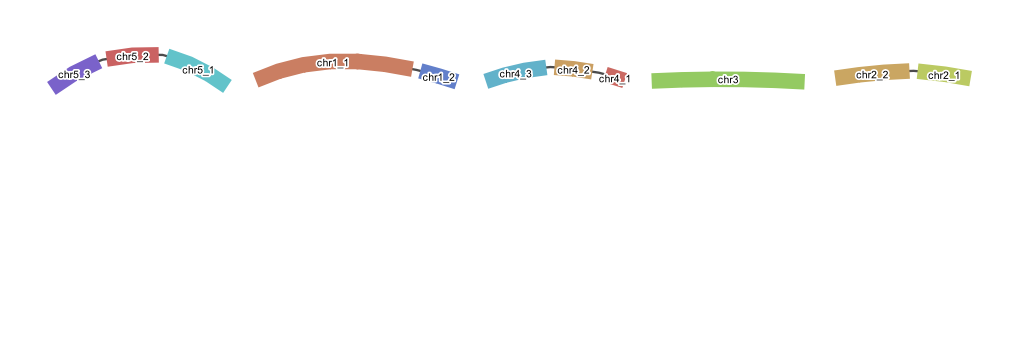


Arabidopsis simulated data: EndHiC cluster


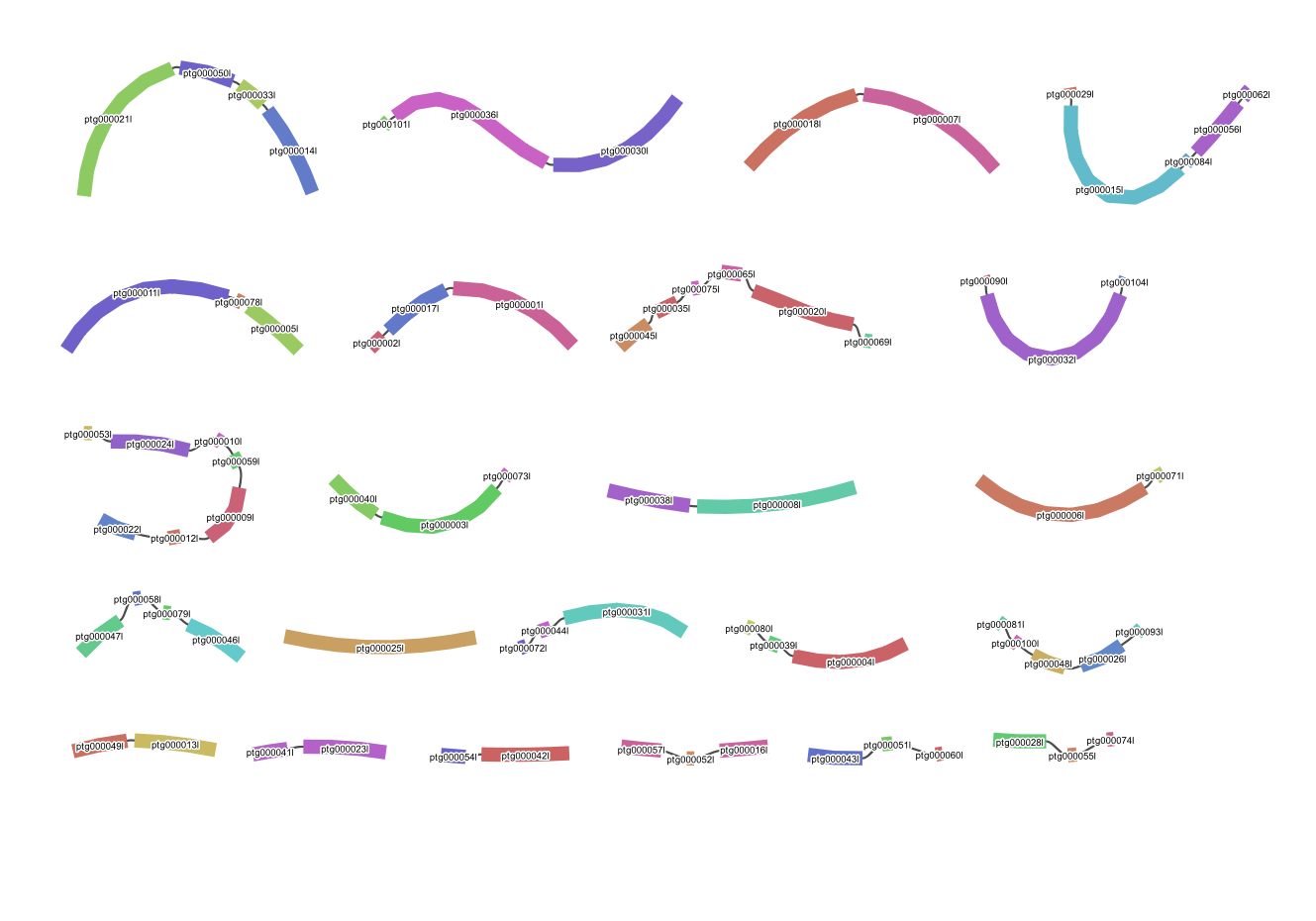


Human real data: EndHiC cluster


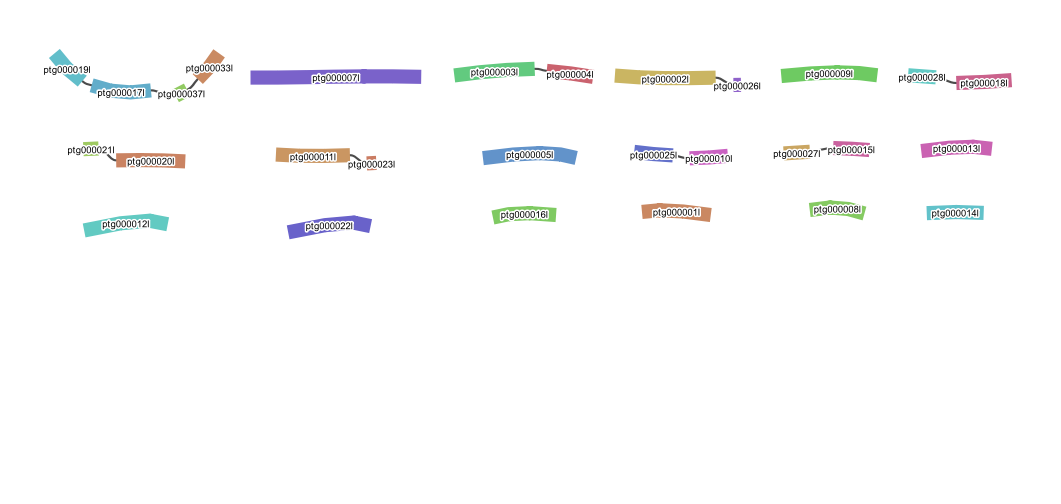


Great burdock real data: EndHiC cluster


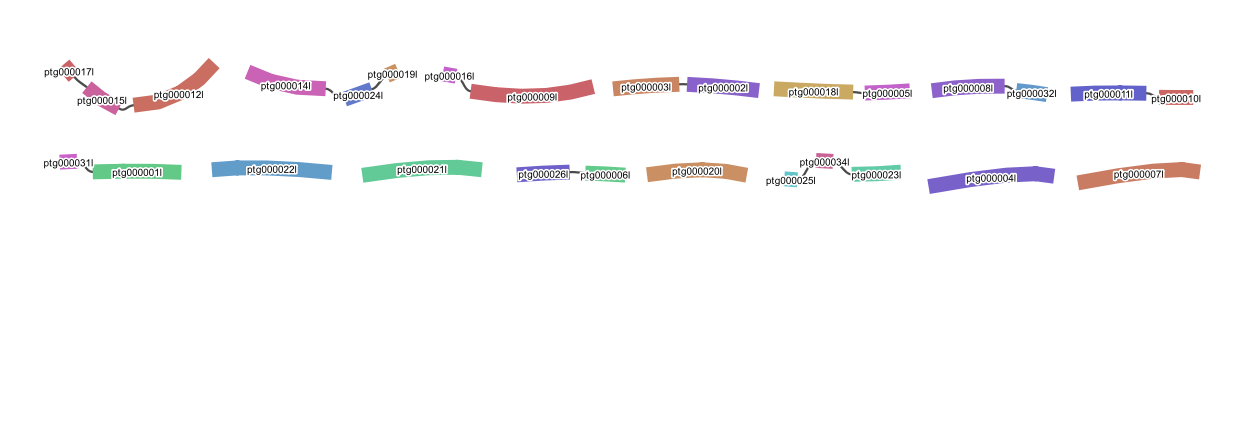


Water spinach real data: EndHiC cluster

**Figure S4.** Bandage view of the merged chromosome-level consensus scaffolds (clusters) including contig order and orientation, produced by running EndHiC on three simulated (human, rice, Arabdopsis) and three real (human, great burdock, water spinach) datasets. In the simulated data, each chromosome of reference genome was randomly split into 1 to 6 contigs, and EndHiC has anchored 100% of the contigs into chromosomes; while in the real data, the contigs were assembled by hifiasm with real PacBio HiFi reads, and EndHiC has anchored 99.3%, 99.8%, 99.7% of contigs into chromosomes for human, great burdock, and water spinach, respectively. Note that all the used Hi-C sequencing reads were real data downloaded from public database.


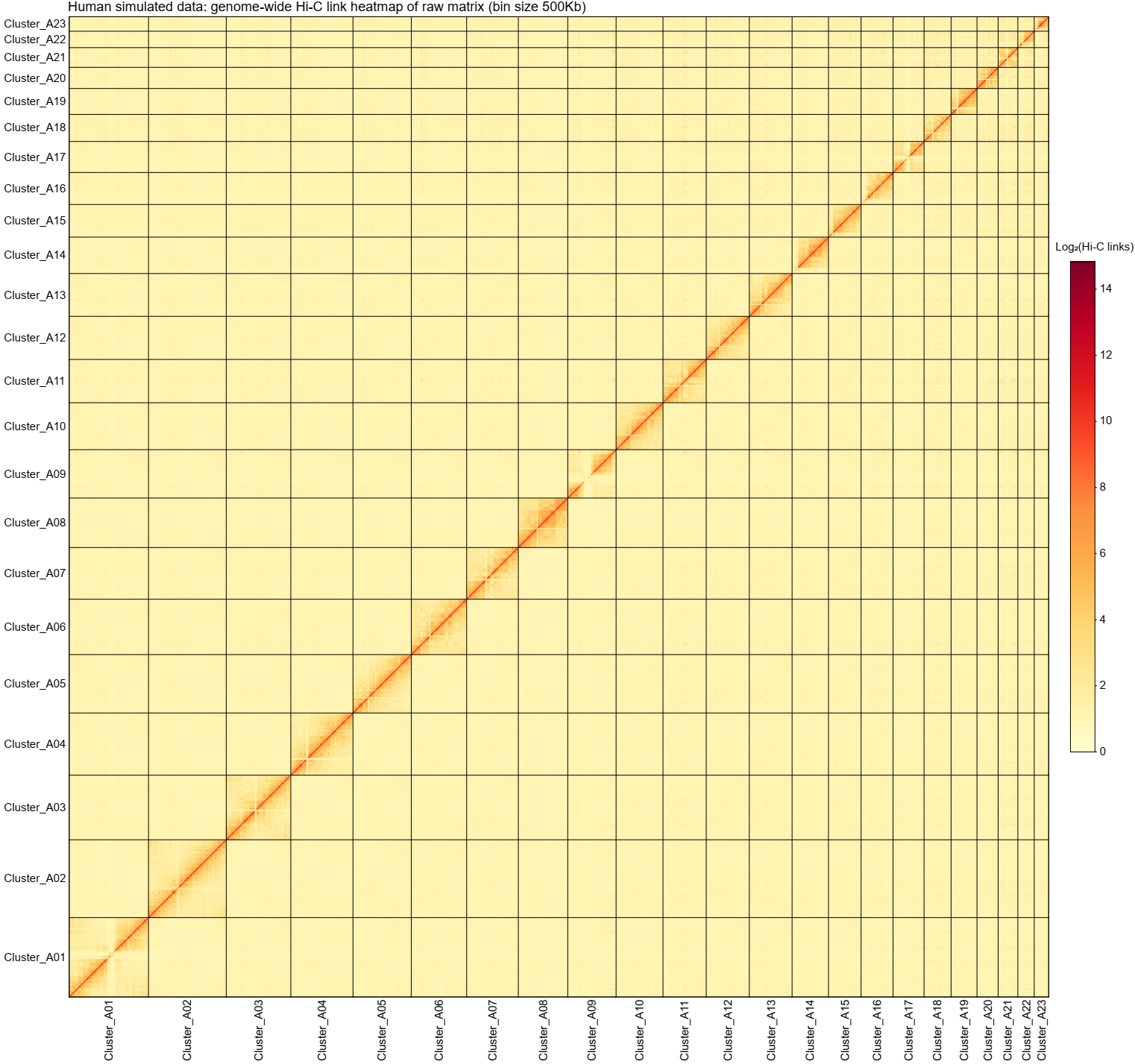


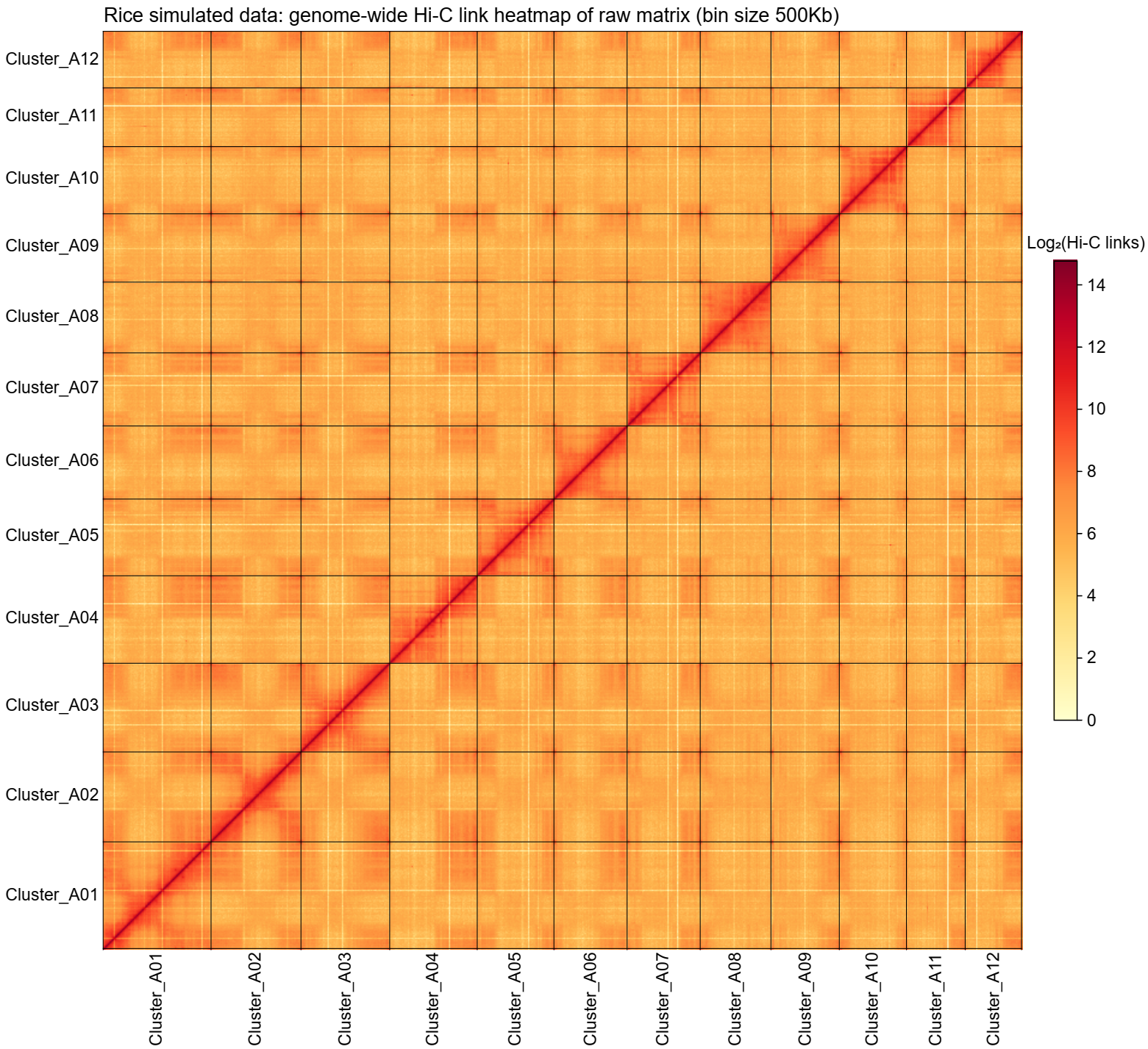


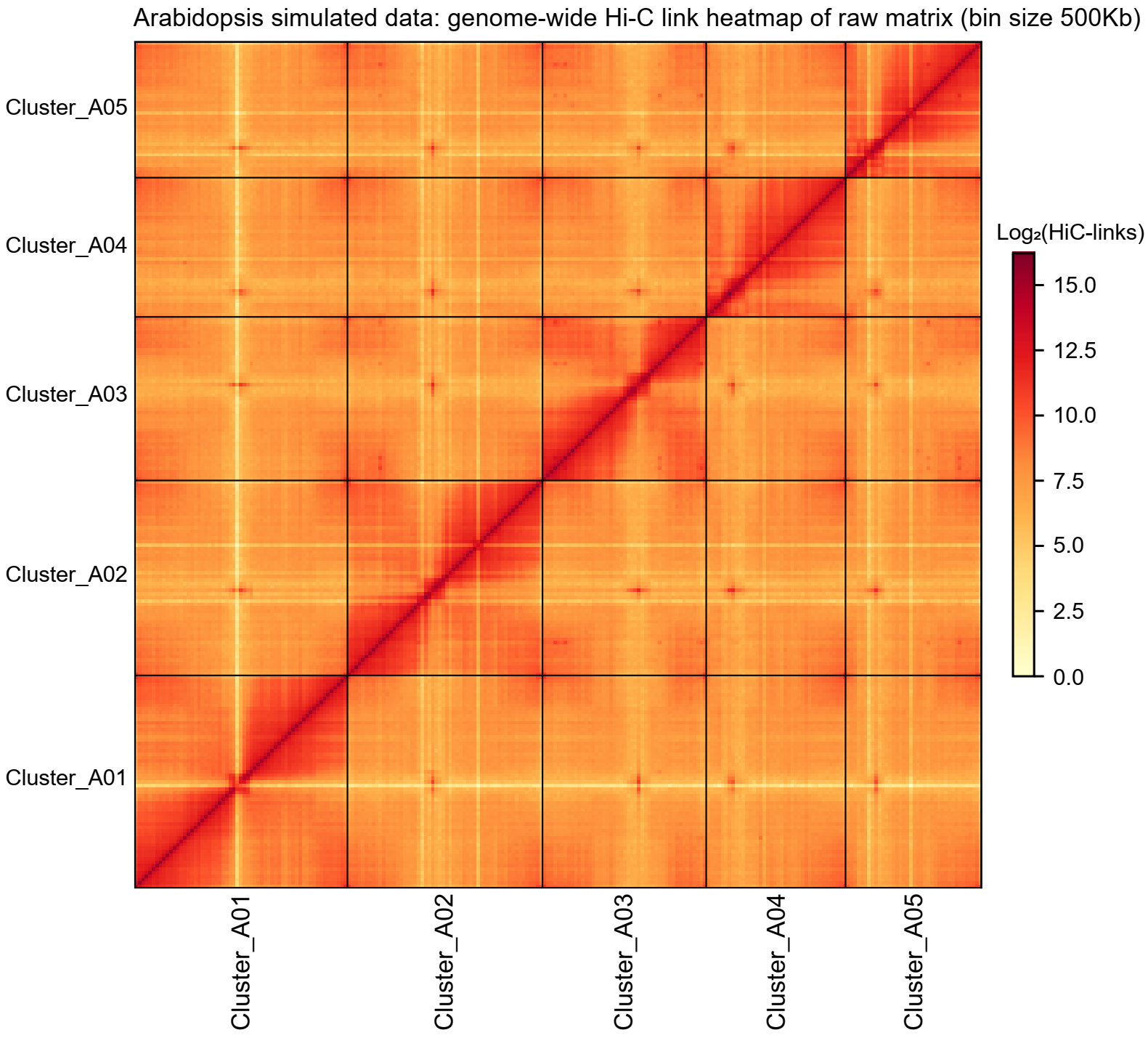


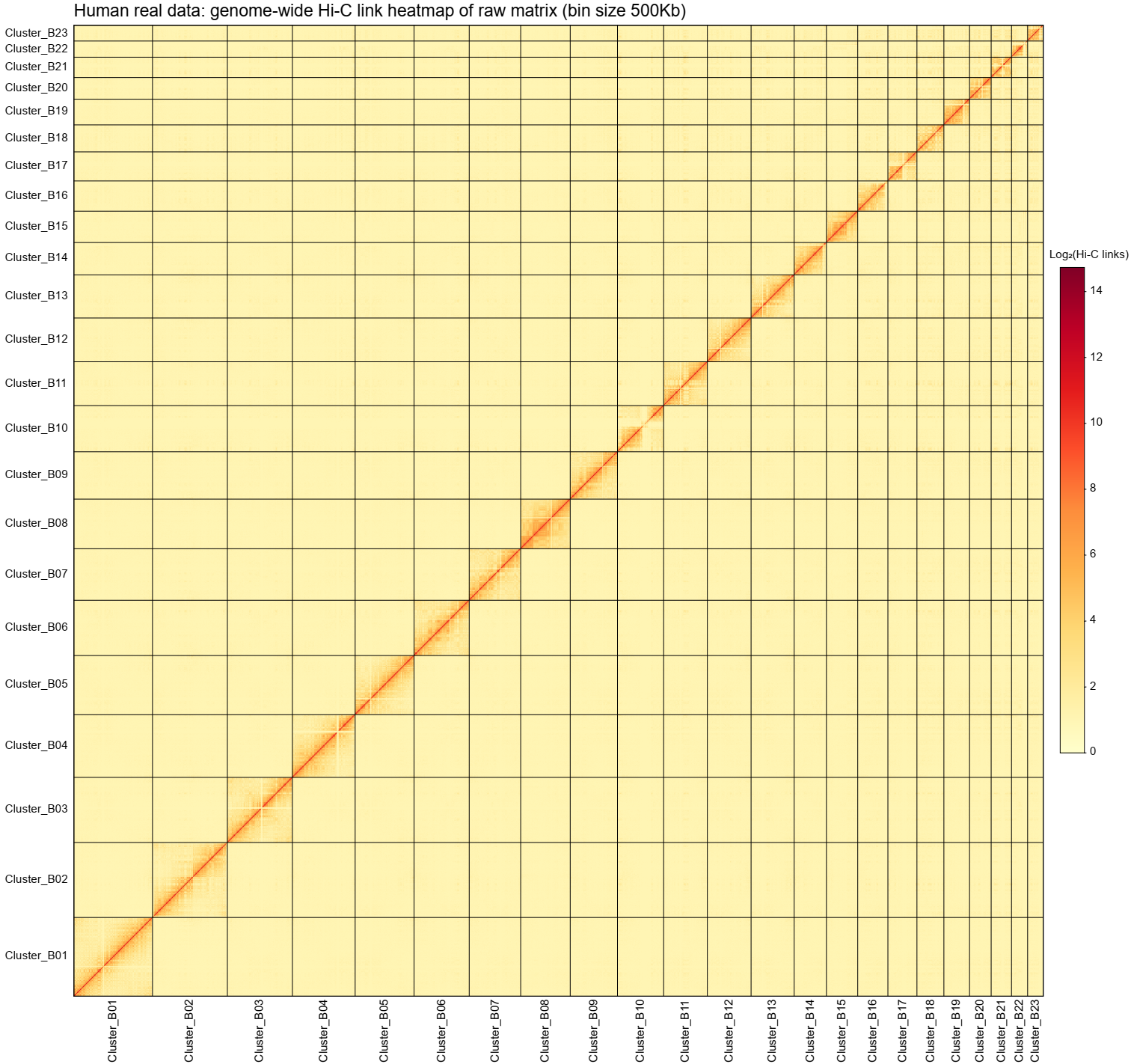


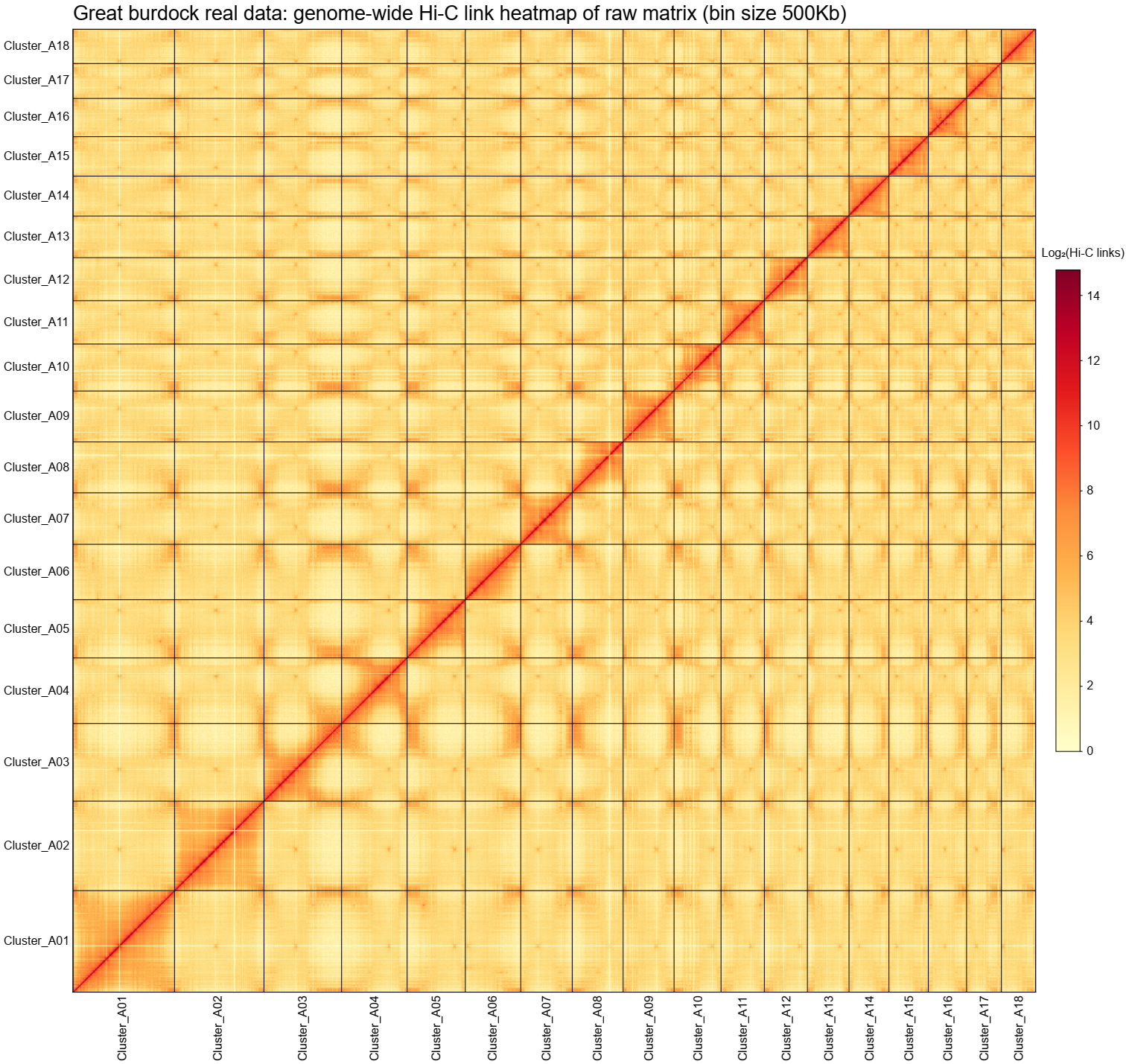


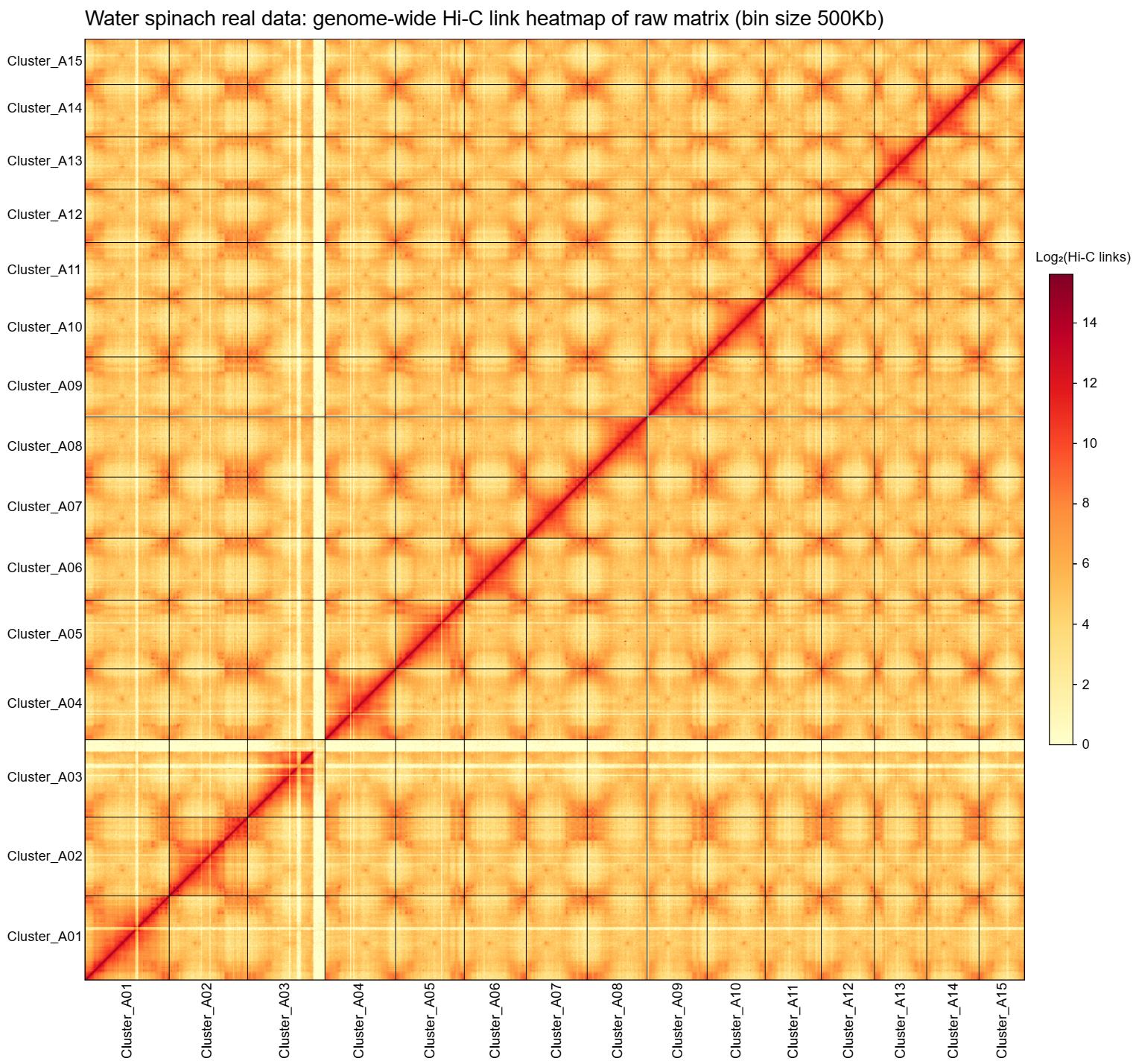


**Figure S5.** Genome-wide Hi-C link heatmap of the merged chromosome-level consensus scaffolds (clusters), produced by running EndHiC on three simulated (human, rice, Arabdopsis) and real (human, great burdock, water spinach) datasets. In the heatmap, each scaffold refers to a pseudo-chromosome, each pixel refers to a 500-Kb bin, and the color value indicates the base 2 logarithm of the number of valid read pairs (log2 [valid read pairs]). The plant genomes (rice, Arabidopsis, great burdock, water spinach) have more inter-chromosome Hi-C interactions than the human genome. In overall, the Hi-C link heatmap style of the simulated datasets is similar to that of real datasets.


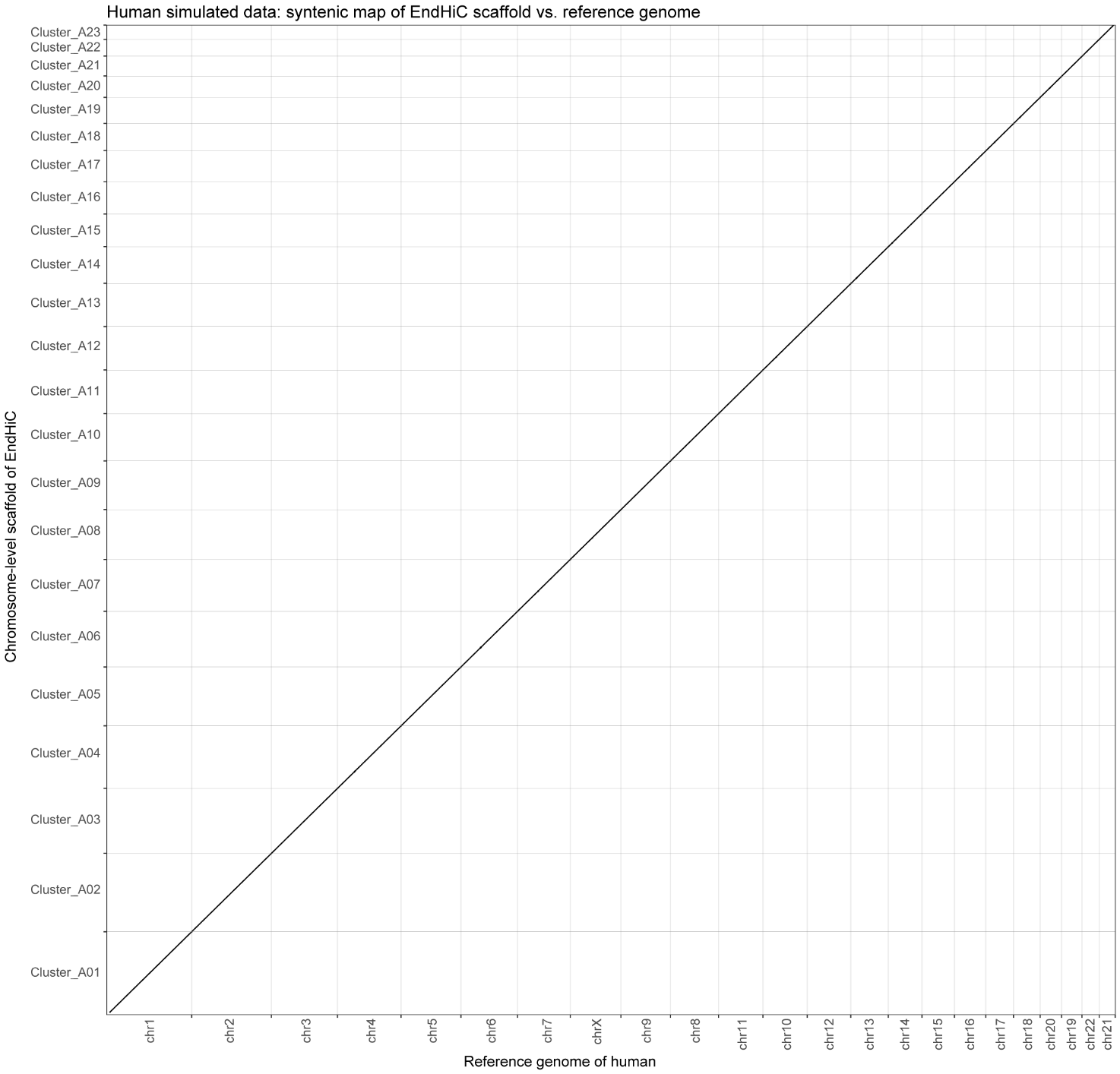


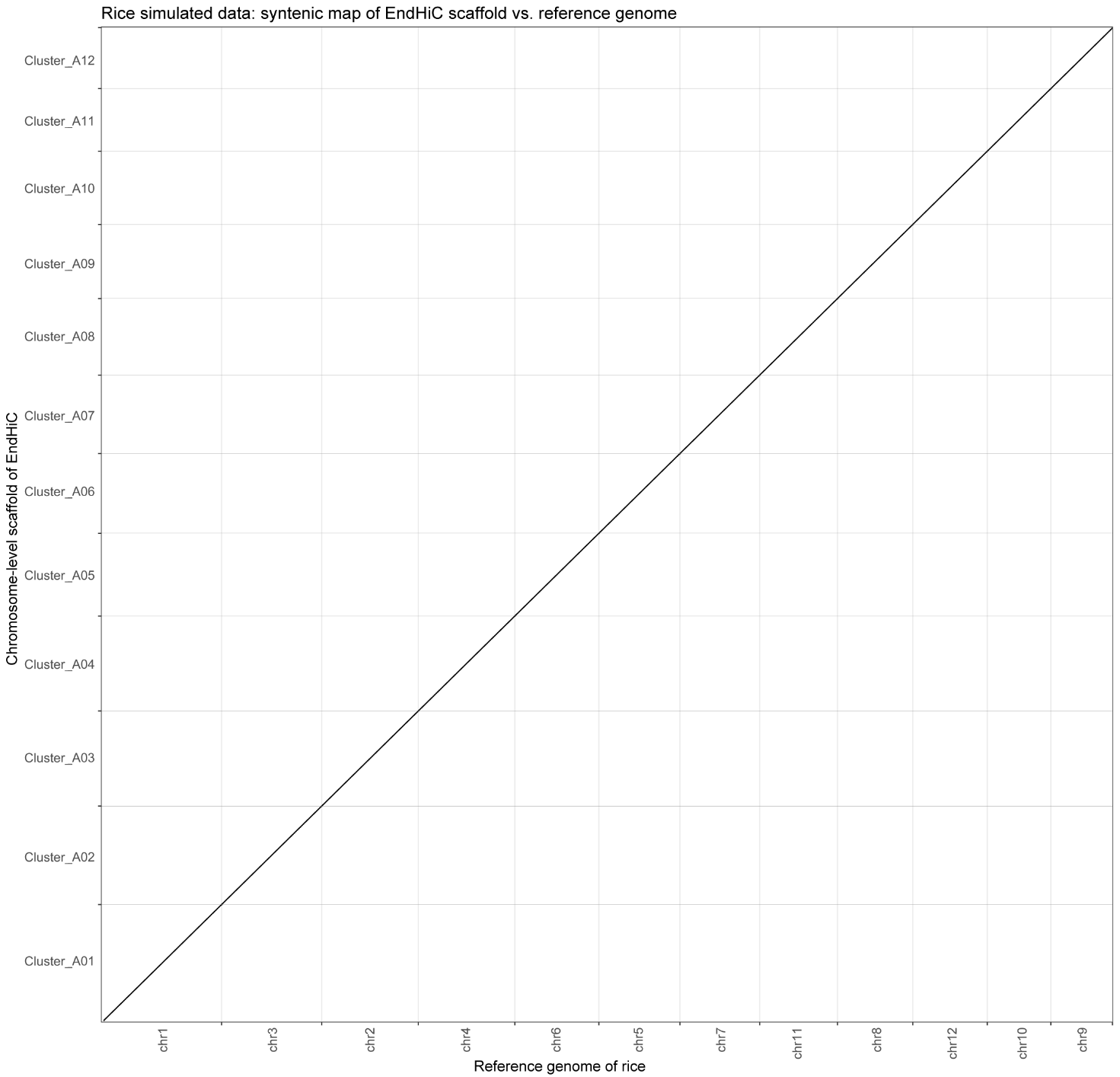


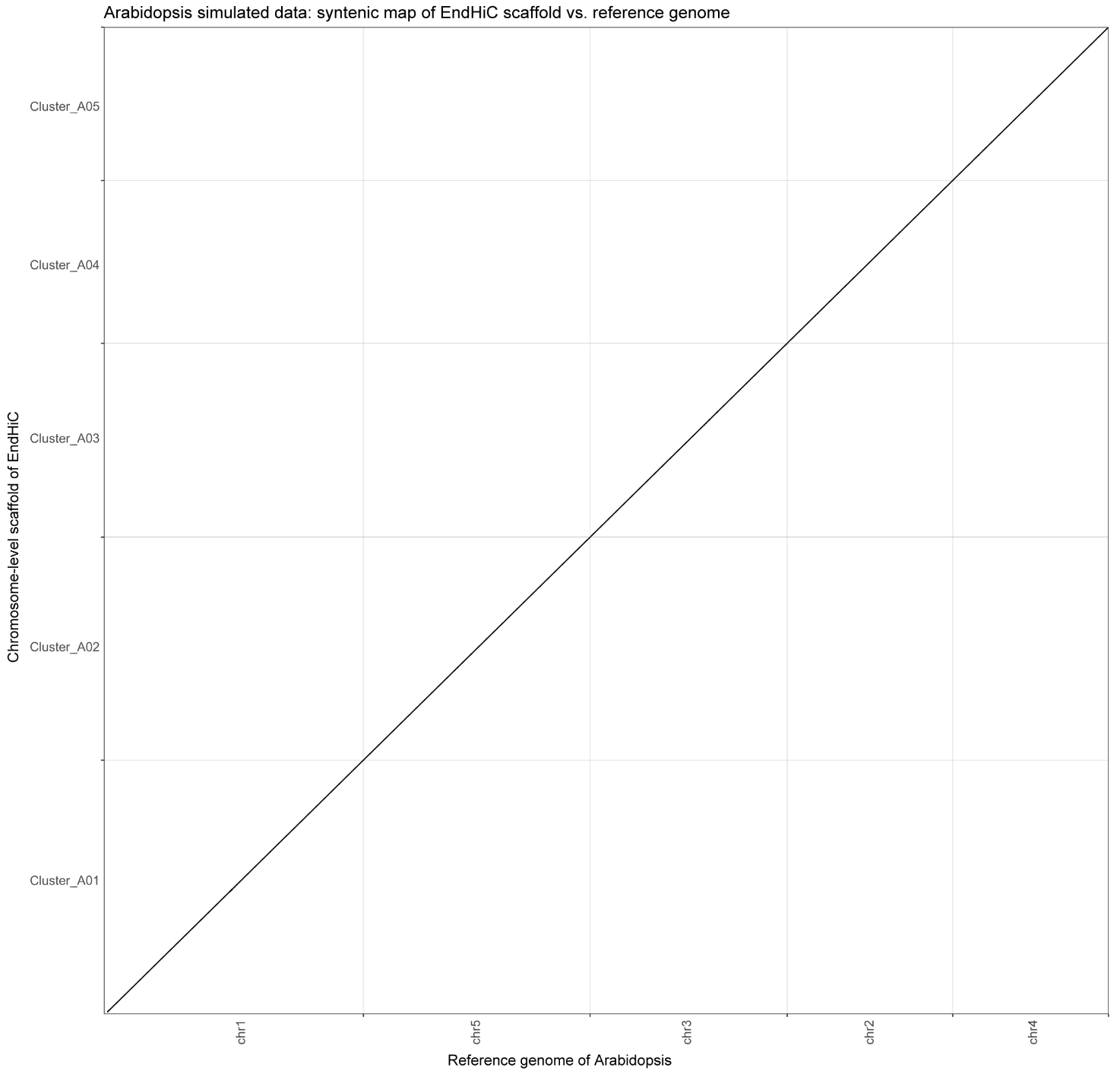


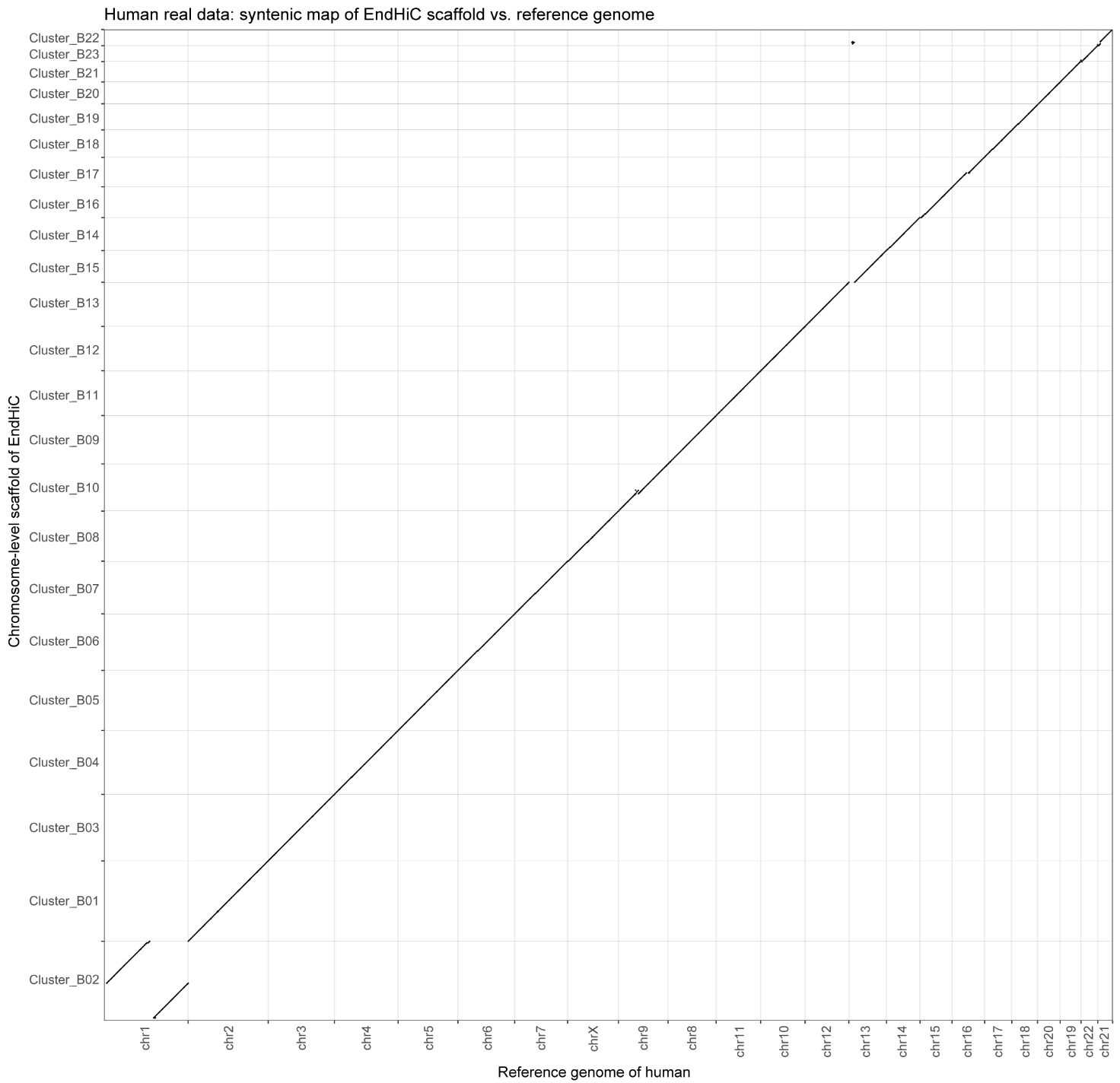


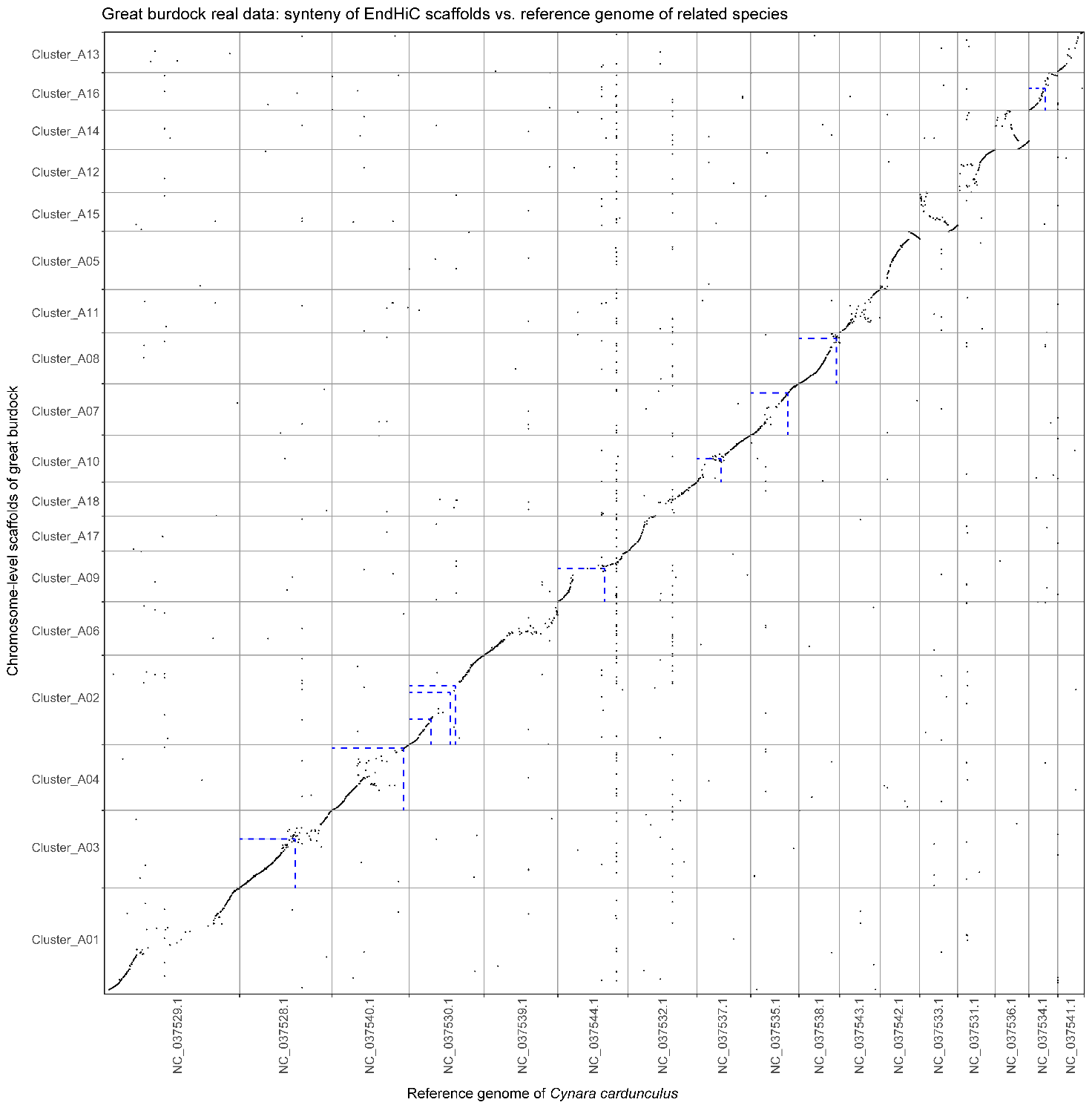


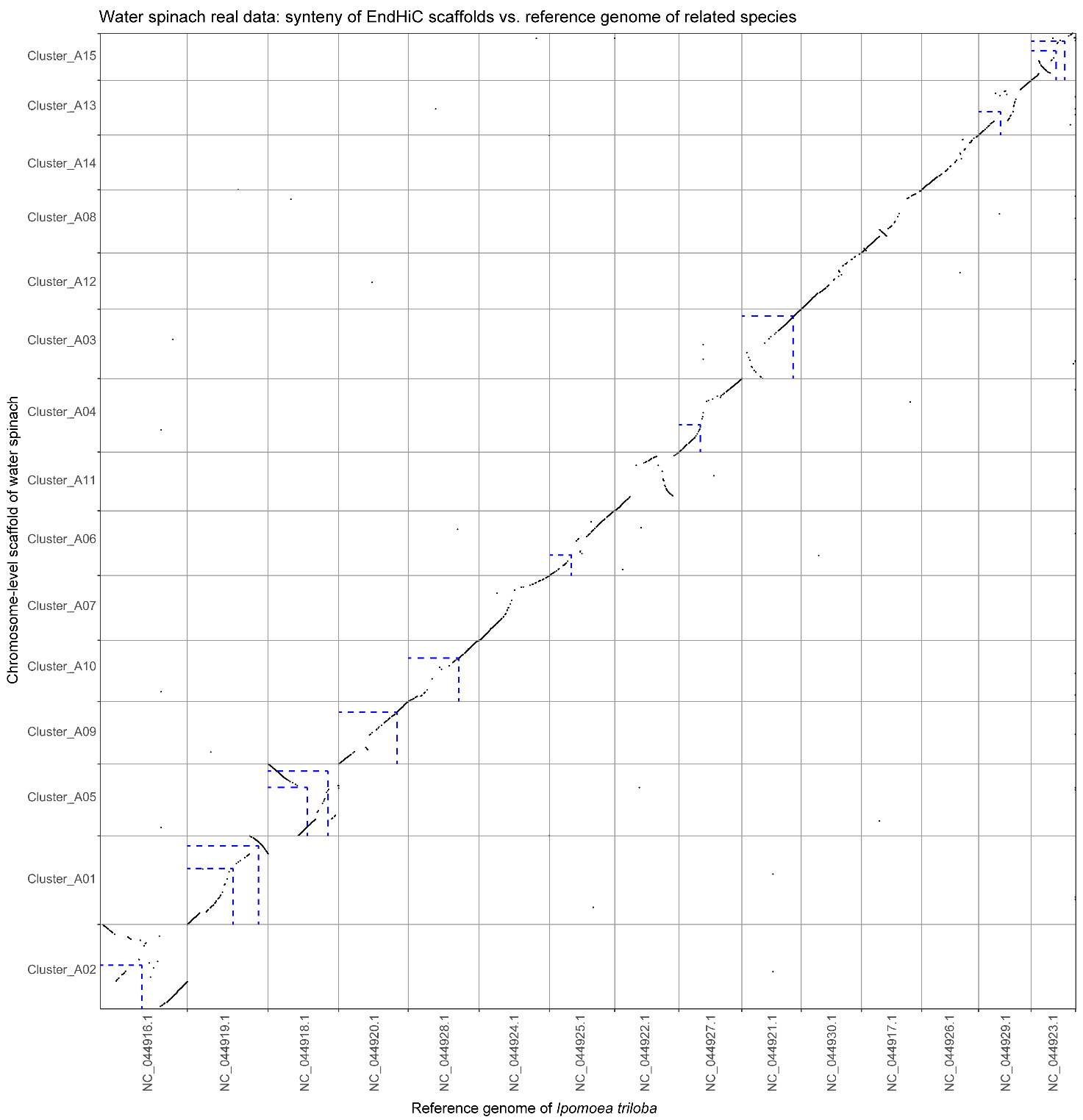


**Figure S6.** Syntenic map showing the whole-genome alignments of the merged chromosome-level consensus scaffolds (clusters) produced by running EndHiC to the reference genomes. The results for three simulated datasets (human, rice, Arabdopsis) and three real datasets (human, great burdock, water spinach) were shown. The corresponding reference genome of the same species (human, rice, Arabidopsis) were used for comparison, or the reference genome of closely related species (*Cynara cardunculus* and *Ipomoea triloba*) were used if the reference genome of the same species is not available. Note that great burdock has 18 chromosomes (haploid) and *C. cardunculus* has 17 chromosomes, 16 chromosomes of the former and 16 of the latter are one-to-one, except that Cluster_A17 and Cluster_A18 of burdock map to the same chromosome (NC_037532.1) of *C.cardunculus*; water spinach and *I. triloba* both have 15 chromosomes which are mapped in one-to-one manner. The whole genome alignment was performed by minimap2, and then the synteny map was drawn by dotPlotly (<https://github.com/tpoorten/dotPlotly>). The simulated datasets all have perfect syntenic relationships with the reference genomes. For the human real dataset, only two contigs of chromosome 1 is mis-ordered, due to the extremely long heterochromatin (~20 Mb) between them. For the real datasets of great burdock and water spinach, the syntenic relationships with their closely related species is not perfect, but largely consistent, which is reasonable considering their divergence time. In addition, the blue dashed lines were used to mark the contig border in the scaffolds, and most syntenic breakpoints were not consistent with the contig separation points, confirming that these syntenic breakpoints were not caused by mis-scaffolding.


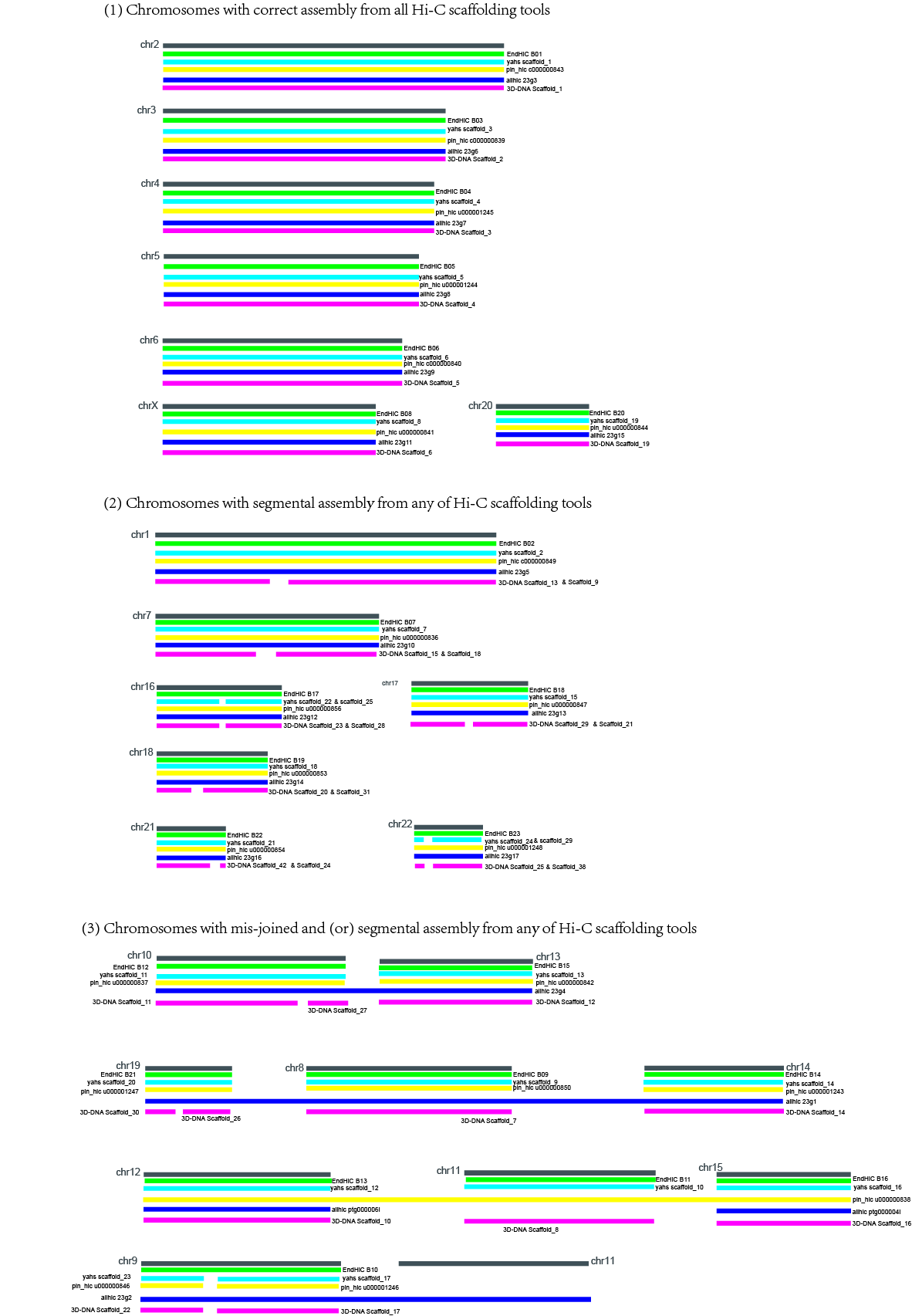


**Figure S7.** Comparison of scaffolding results for the human real data from LACHESIS, AllHiC, 3D-DNA, Pin_hic and YaHS. Each color represents for a different scaffolding tool. There are 7 chromosomes (chr2, chr3, chr4, chr5, chr6, chrX, chr20) that have been successfully scaffolded by all the scaffolding tools, 7 chromosomes (chr1, chr7, chr16, chr17, chr18, chr21, chr22) that have been only successfully scaffolded by EndHiC but segmental scaffolded by one or more of the other scaffolding tools, and 9 chromosomes (chr10, chr13, chr19, chr8, chr14, chr12, chr11, chr15, chr9) that have been only successfully scaffolded by EndHiC but mis-joining and (or) segmental scaffolded by one or more of the other scaffolding tools. Note that LACHESIS results were not shown, because LACHESIS mis-joined all the 23 human chromosomes into one scaffold.


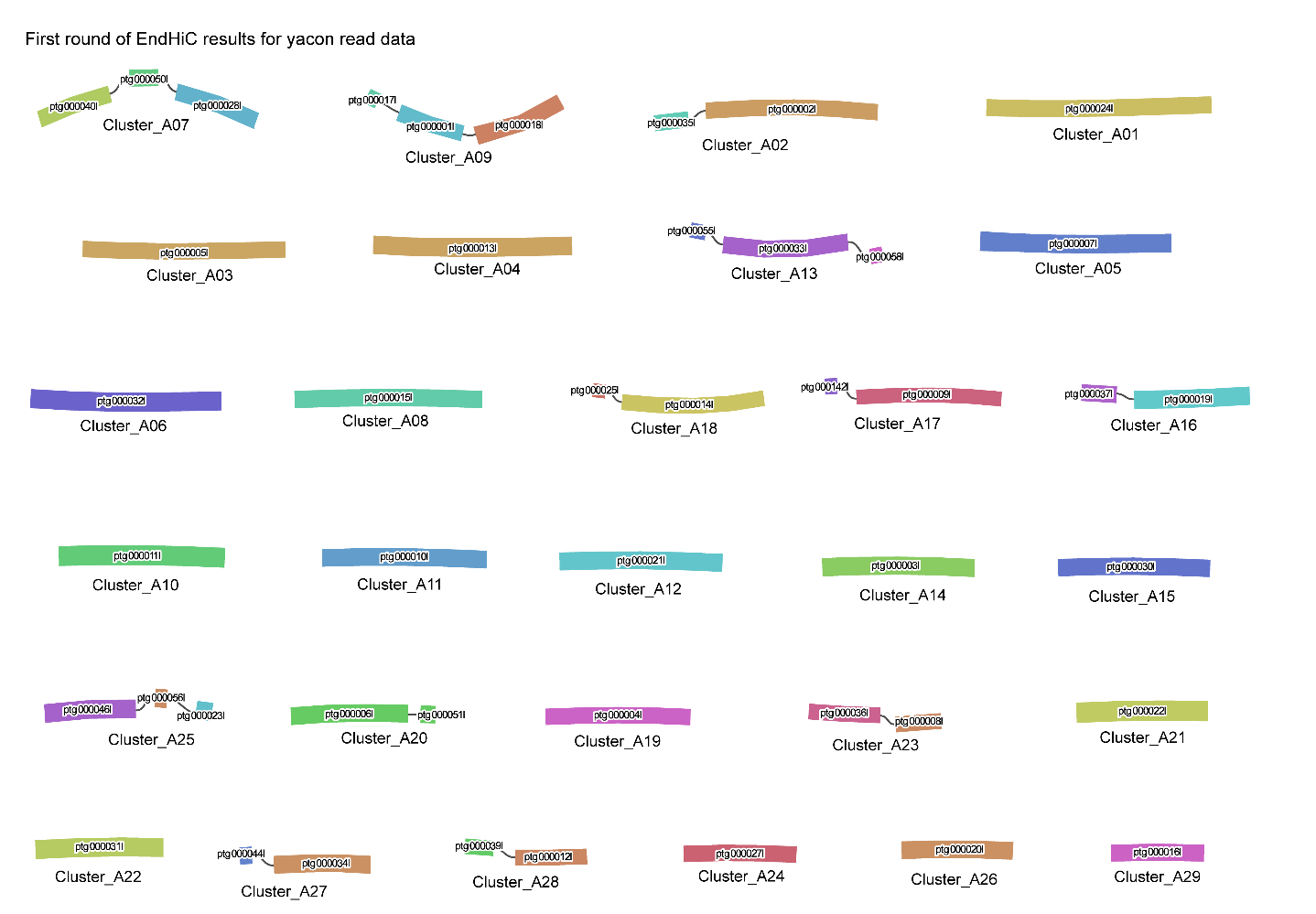


**Figure S8**. Bandage view of EndHiC results for yacon (*Smallanthus sonchifolius*) real data. In the first round of EndHiC results, all the contigs (lengths > 1 Mb) are assembled into 29 chromosome-level scaffolds (prefix Cluster_A).


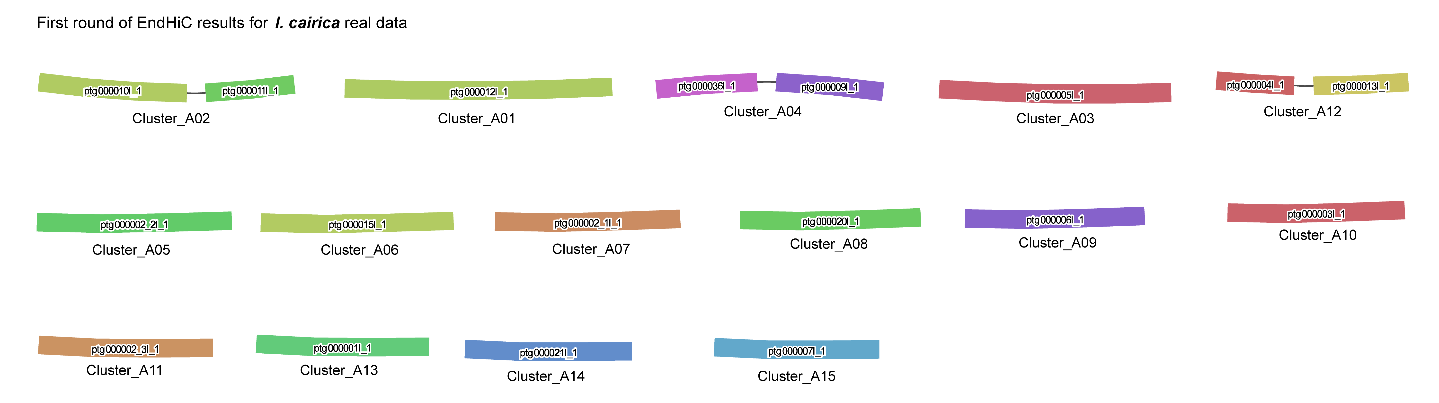


**Figure S9**. Bandage view of EndHiC results for *Ipomoea cairica* real data. In the first round of EndHiC results, all the contigs (lengths > 1 Mb) are assembled into 15 chromosome-level scaffolds (prefix Cluster_A).


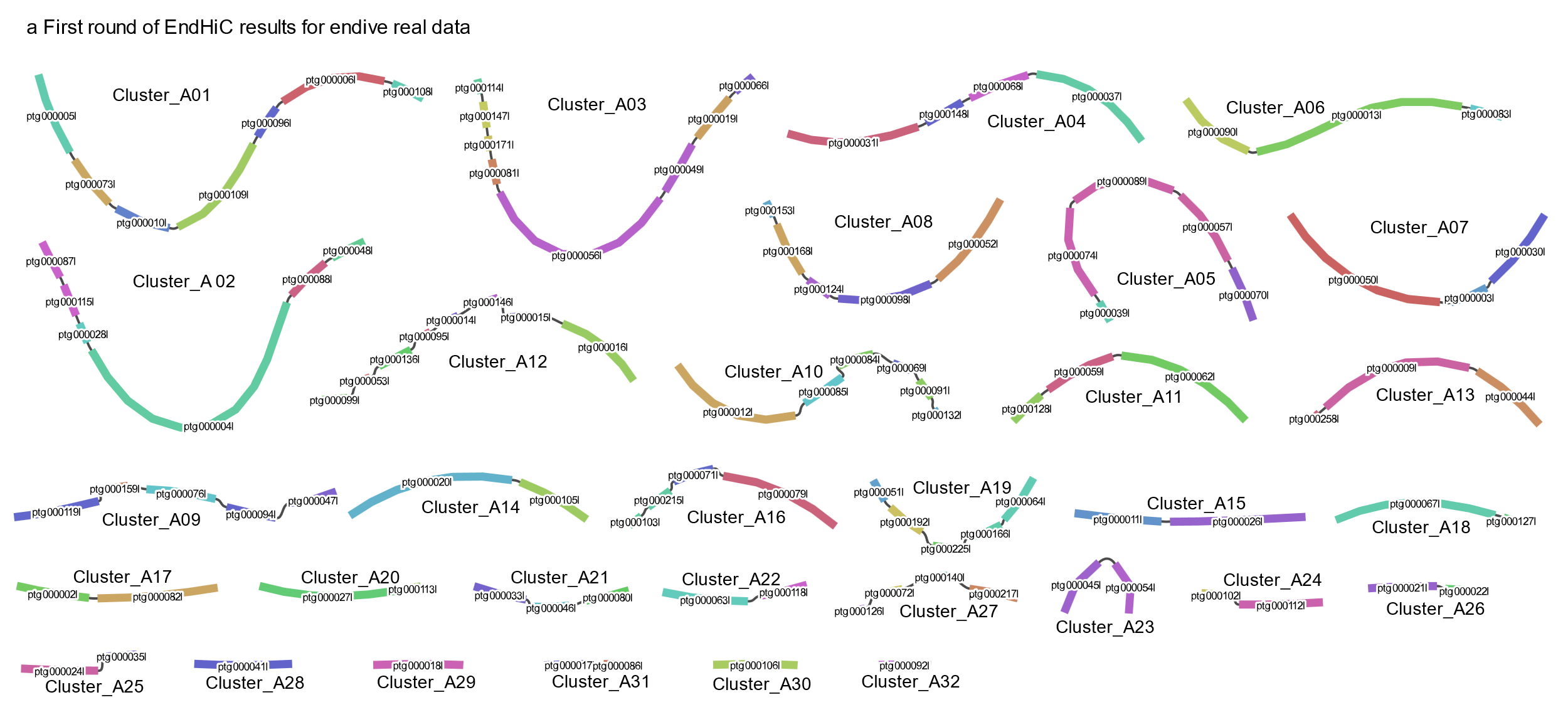


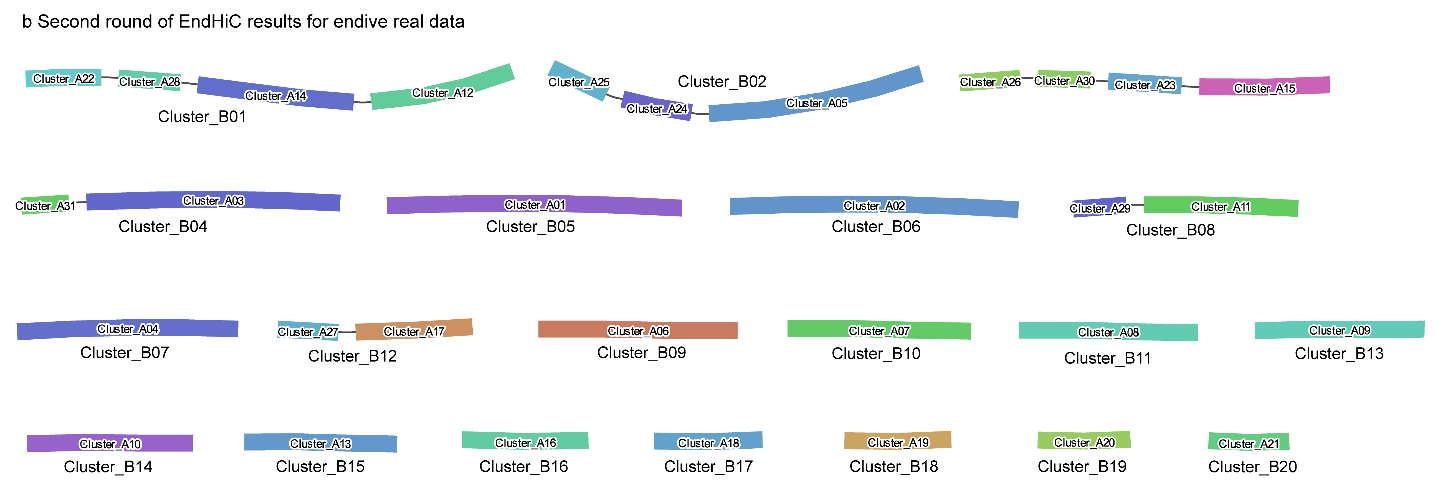


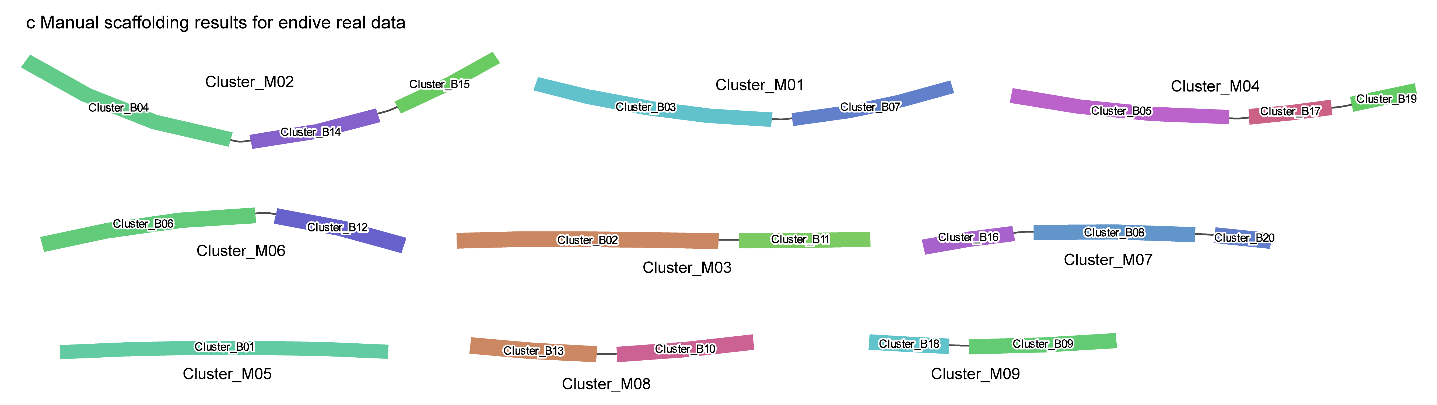


**Figure S10**. Bandage view of EndHiC results for endive (*Cichorium endivia*) real data. **a.** In the first round of EndHiC results, all the contigs (lengths > 1 Mb) are assembled into 32 scaffolds (prefix Cluster_A). **b.** In the second round of EndHiC results, all the scaffolds from the first round are assembled into 20 scaffolds (prefix Cluster_B). **c.** All the scaffolds from the second round are manually assembled into 9 chromosome-level scaffolds (prefix Cluster_M).


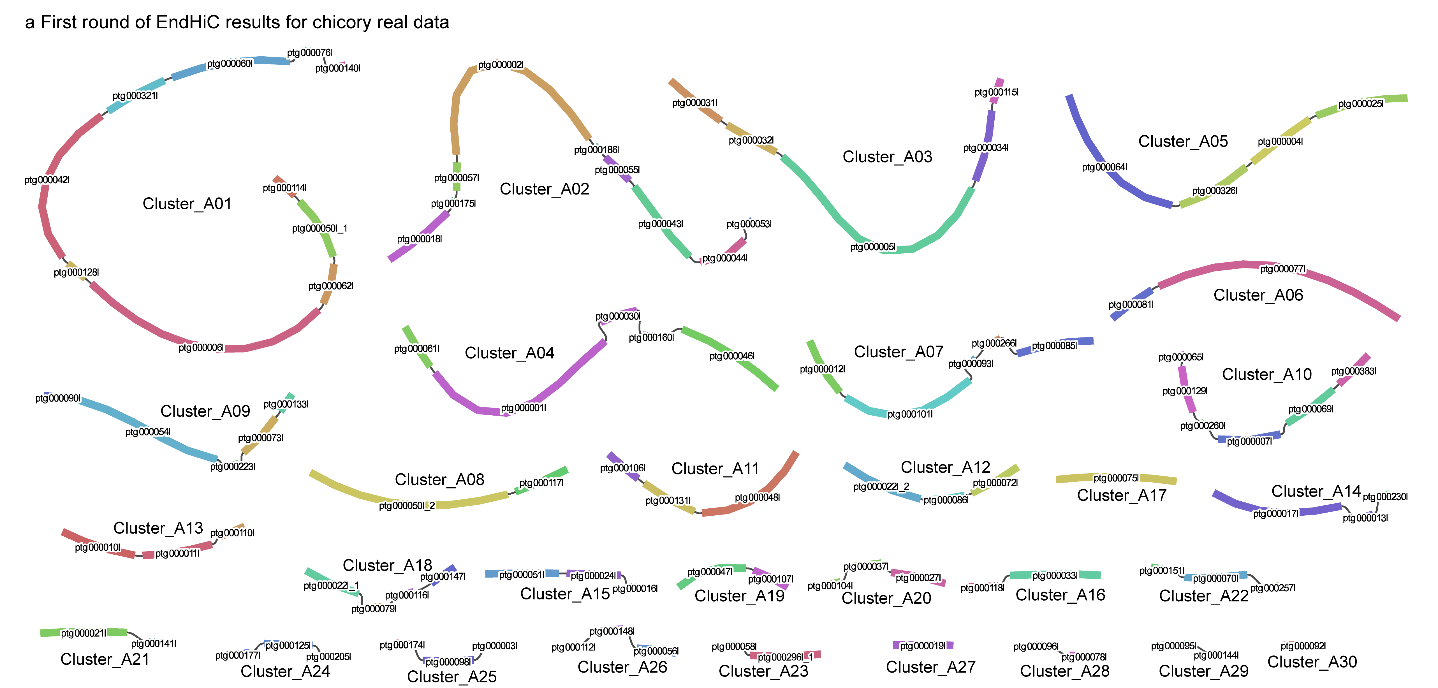


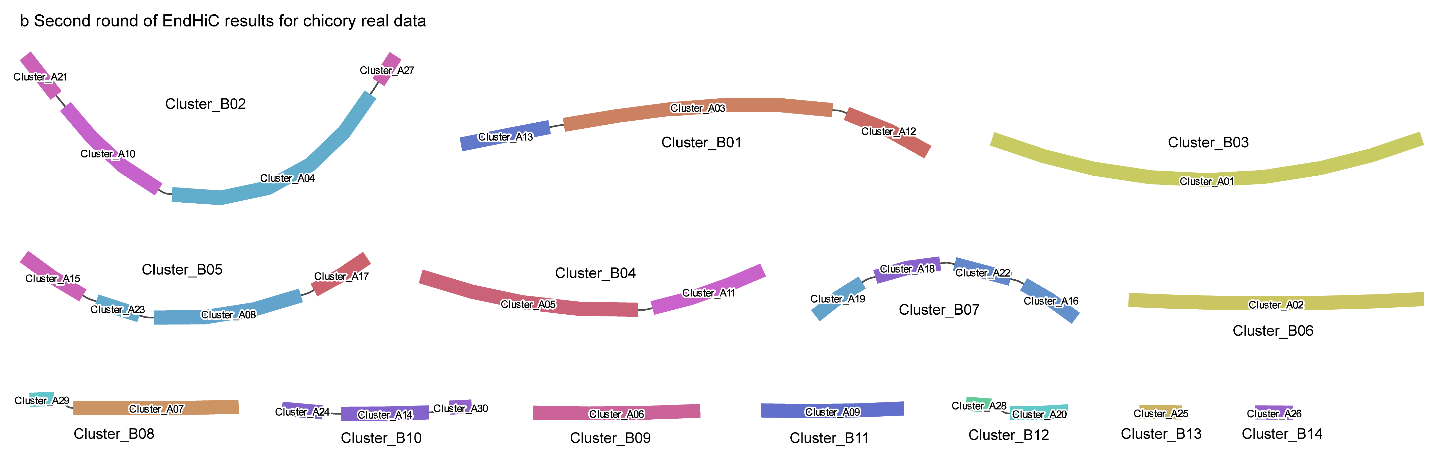


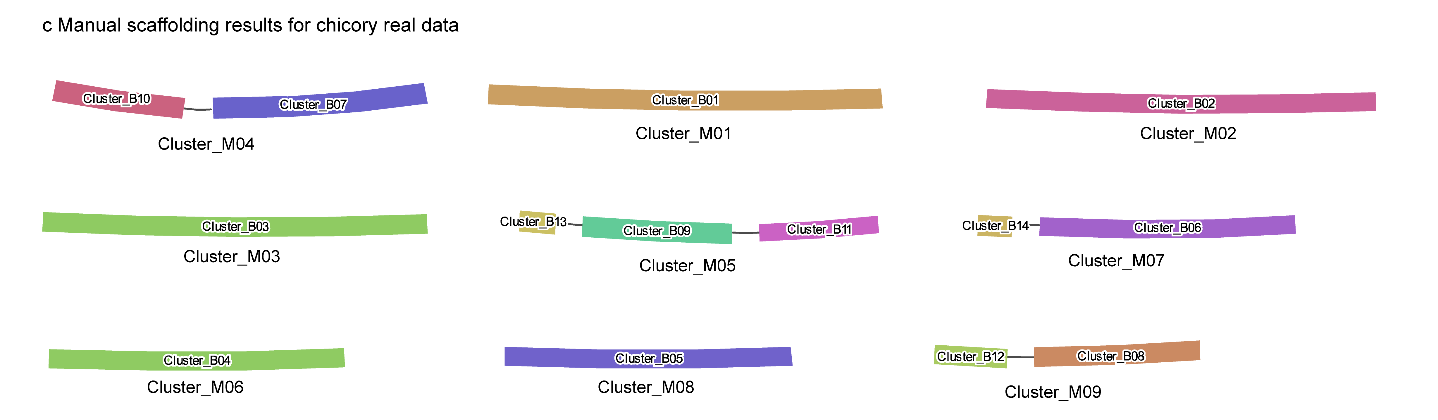


**Figure S11**. Bandage view of EndHiC results for chicory (*Cichorium intybus*) real data. **a.** In the first round of EndHiC results, all the contigs (lengths > 1 Mb) are assembled into 30 scaffolds (prefix Cluster_A). **b.** In the second round of EndHiC results, all the scaffolds from the first round are assembled into 14 scaffolds (prefix Cluster_B). **c.** All the scaffolds from the second round are manually assembled into 9 chromosome-level scaffolds (prefix Cluster_M).


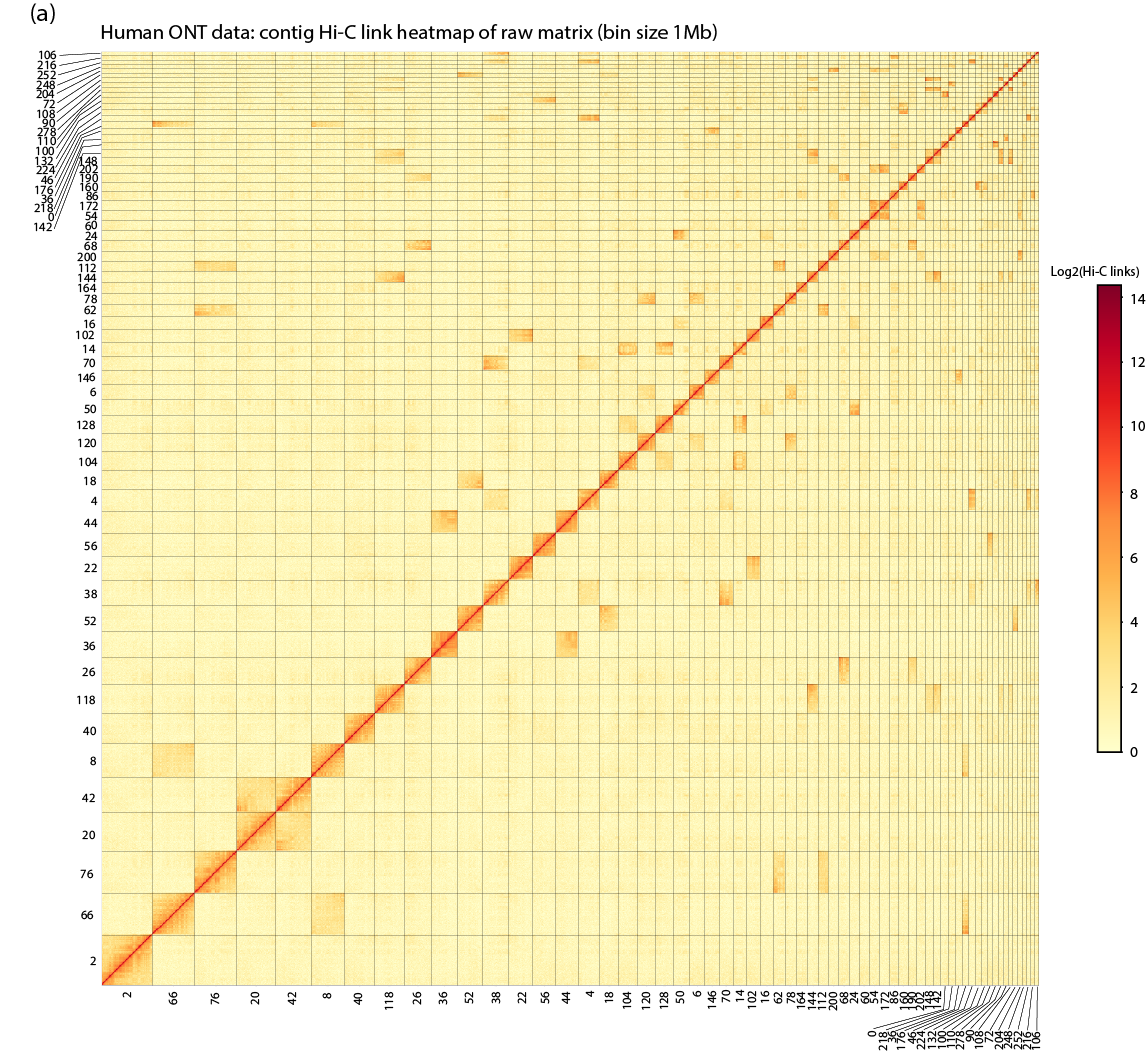


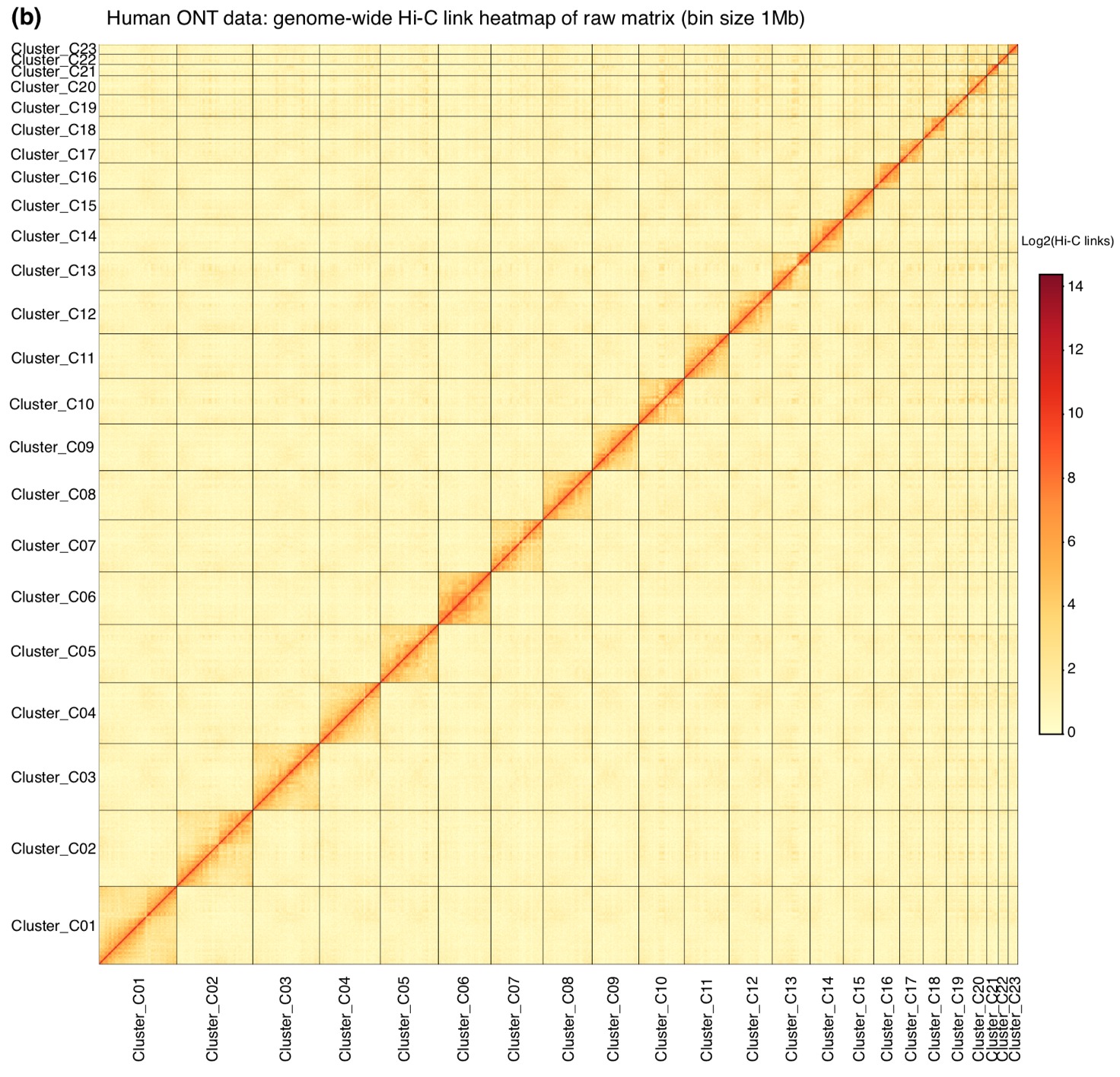


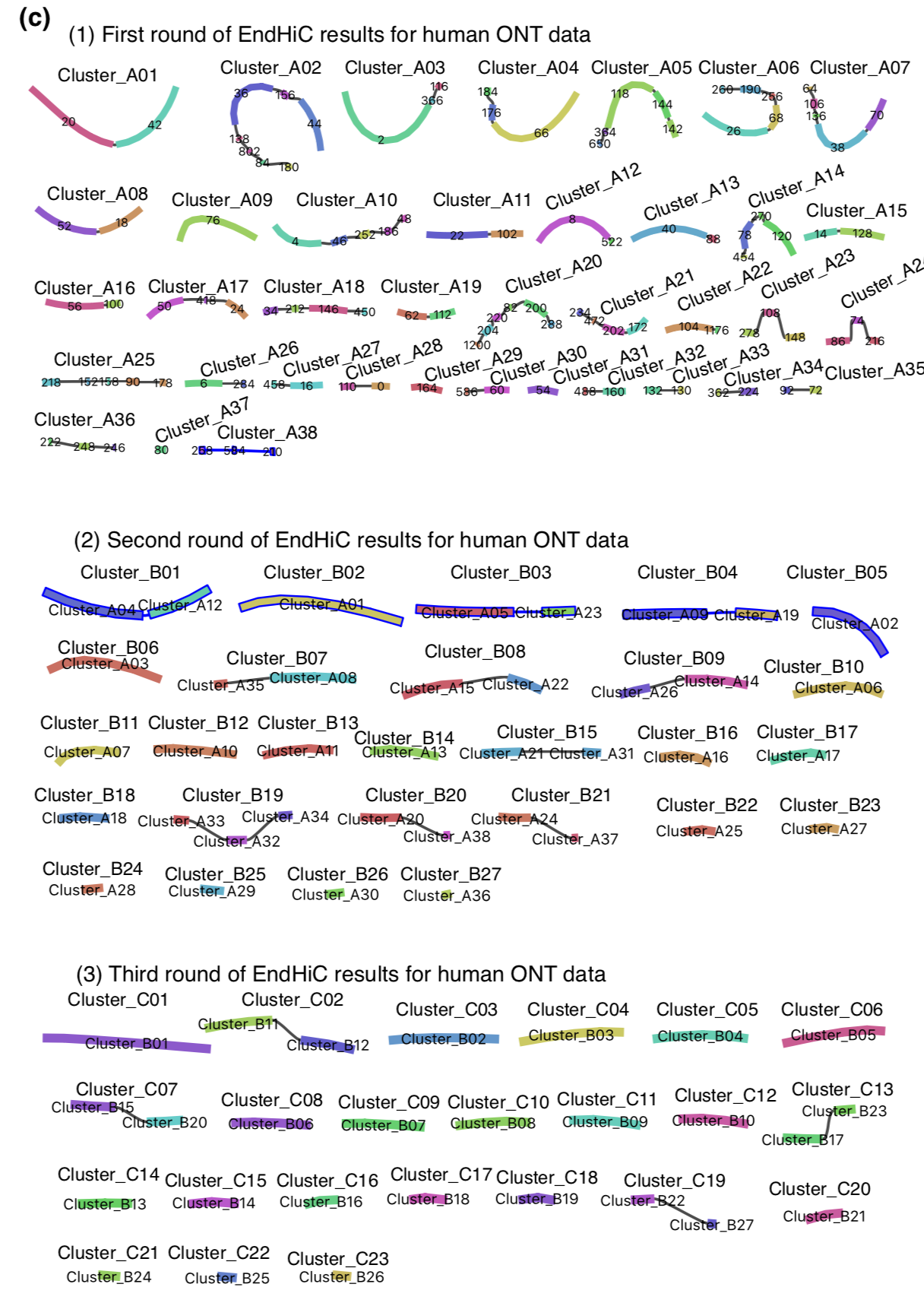


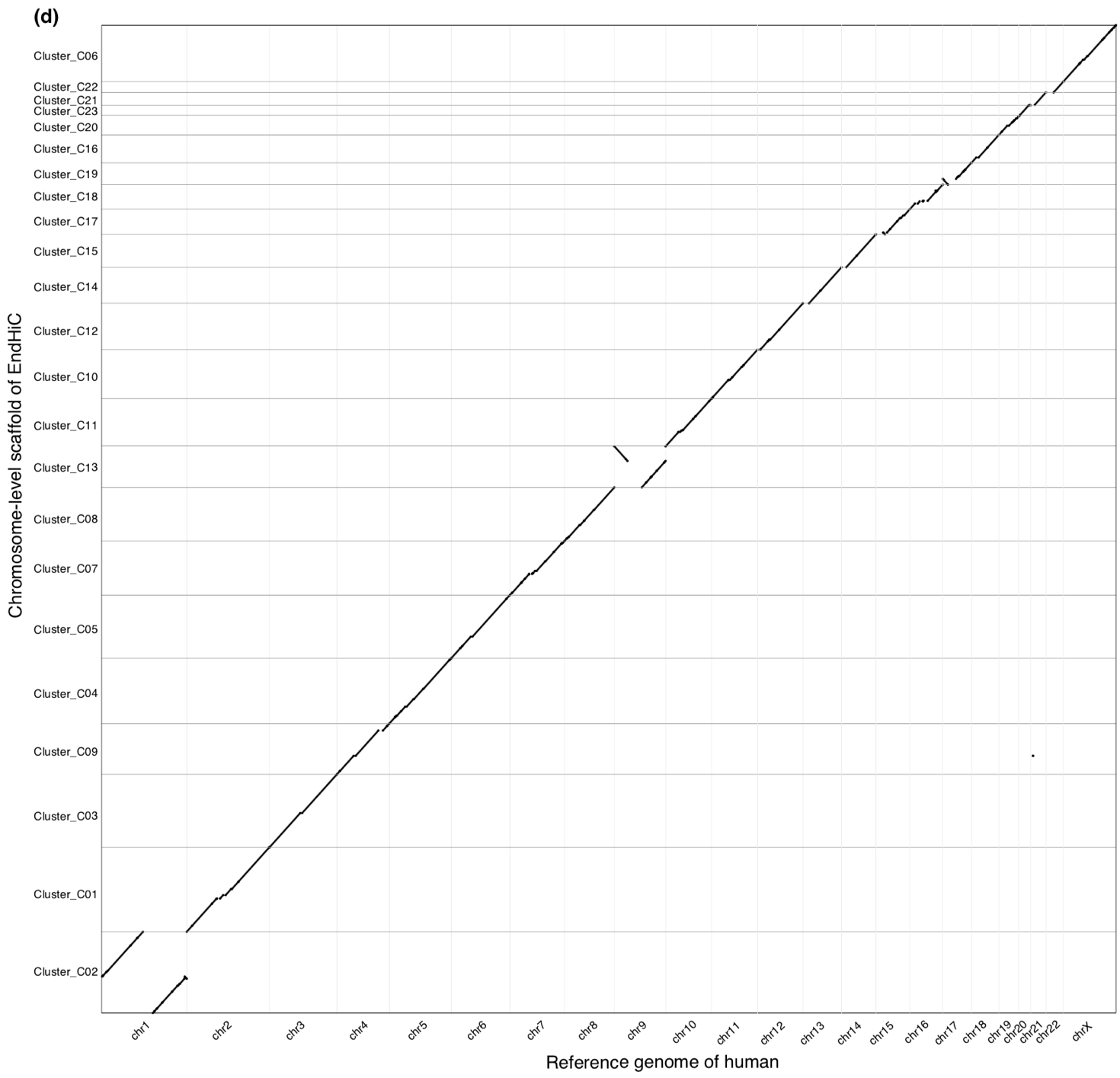


**Figure S12**. Analysis results for EndHiC on the human ONT data. **a** Hi-C link heatmap of the ONT-assembled contigs. The contigs with the length less than 10 Mb were not shown, which takes less than 10% of the total sequence length. Each pixel refers to a 1Mb bin, and the color value indicates the base 2 logarithm of the number of valid read pairs (log2 [valid read pairs]). **b** Hi-C link heatmap of the final EndHiC scaffolds. In the heatmap, each scaffold refers to a pseudo-chromosome, each pixel refers to a 1Mb bin. **c** Bandage view of EndHiC results. The first round of EndHiC results using contig end sizes 0.5-Mb to 2.5-Mb, all the contigs are assembled into 38 scaffolds (prefix Cluster_A); the second round of EndHiC results using contig end sizes 3.0-Mb to 5.0-Mb, all the scaffolds from the first round are assembled into 27 scaffolds (prefix Cluster_B); The third round of EndHiC results using contig end sizes 5.5-Mb to 7.5-Mb, all the contigs are assembled into 23 scaffolds (prefix Cluster_C). **d** Syntenic map showing the whole-genome alignments of the final EndHiC scaffolds (clusters) to the reference human genome. Three chromosomes (Chr1, Chr9 and Chr17) have some contigs mis-ordered or mis-orientated, due to the missing large fragments by ONT contig assembly. The whole genome alignment was performed by minimap2, and then the synteny map was drawn by dotPlotly (<https://github.com/tpoorten/dotPlotly>).

**Table S1.** Distribution of signal and noise contact values using raw links data from great burdock

| Contig end(Mb) | Signal (mean) | Noise (mean) | Ratio | Signal (median) | Noise (median) | Ratio | Signal (max) | Noise (max) | Ratio | Signal (min) | Noise (min) | Ratio |
| --- | --- | --- | --- | --- | --- | --- | --- | --- | --- | --- | --- | --- |
| 0.2 | 1,092 | 27 | 41 | 1,161 | 10 | 116 | 2,387 | 189 | 13 | 6 | 1 | 6 |
| 0.5 | 5,892 | 87 | 68 | 6,861 | 17 | 404 | 9,544 | 877 | 11 | 581 | 1 | 581 |
| 1 | 12,662 | 206 | 62 | 14,910 | 64 | 233 | 25,246 | 1,788 | 14 | 995 | 1 | 995 |
| 2 | 27,450 | 541 | 51 | 27,137 | 267 | 102 | 44,836 | 4,485 | 10 | 15,097 | 16 | 944 |
| 5 | 61,609 | 2,379 | 26 | 61,549 | 1,508 | 41 | 87,490 | 18,740 | 5 | 42,986 | 272 | 158 |
| 10 | 124,808 | 7,806 | 16 | 119,619 | 5,538 | 22 | 150,282 | 55,106 | 3 | 103,192 | 1,051 | 98 |
| 20 | 249,197 | 24,174 | 10 | 252,392 | 19,891 | 13 | 308,973 | 138,819 | 2 | 186,227 | 3,587 | 52 |
| 40 | 453,426 | 93,551 | 5 | 491,316 | 62,844 | 8 | 609,217 | 232,258 | 3 | 259,746 | 27,805 | 9 |

Note: The contact values from adjacent contig ends and non-adjacent contig ends are taken as signal and noise contact values, respectively. For gradient contig end sizes 0.2 Mb, 0.5 Mb, 1 Mb, 2 Mb, 5 Mb, 10 Mb, 20 Mb and 40 Mb, the ratio of the mean values, median values, max values, and min values from the signal and noise groups were calculated. All types of the signal to noise ratios showed that the difference between the signal and noise become smaller when the contig end size grows larger.

**Table S2.** Number of the clusters under each parameter combination.

| **Contig end size** | **Type of Hi-C link matrix** | **Times of the turning point as contact cutoff for signal and noise linkages** | | | | | | | | |
| --- | --- | --- | --- | --- | --- | --- | --- | --- | --- | --- |
|  |  | **1** | **1.5** | **2** | **2.5** | **3** | **3.5** | **4** | **4.5** | **5** |
| 500-Kb | Raw | 17 | 19 | 20 | 20 | 20 | 20 | 20 | 20 | 20 |
|  | Normalized | 15 | 18 | 19 | 19 | 19 | 19 | 19 | 19 | 19 |
| 1.0-Mb | Raw | 17 | 18 | 19 | 20 | 20 | 20 | 20 | 20 | 20 |
|  | Normalized | 17 | 18 | 19 | 19 | 19 | 19 | 19 | 19 | 19 |
| 1.5-Mb | Raw | 17 | 18 | 18 | 18 | 18 | 18 | 18 | 18 | 18 |
|  | Normalized | 16 | 18 | 18 | 18 | 18 | 18 | 18 | 18 | 18 |
| 2.0-Mb | Raw | 17 | 18 | 18 | 18 | 18 | 18 | 18 | 18 | 18 |
|  | Normalized | 17 | 18 | 18 | 18 | 18 | 18 | 18 | 18 | 18 |
| 2.5-Mb | Raw | 17 | 18 | 18 | 18 | 18 | 18 | 18 | 18 | 18 |
|  | Normalized | 17 | 18 | 18 | 18 | 18 | 18 | 18 | 18 | 18 |

Note: This is the summary of all the cluster results from a standard EndHiC run for the great burdock (n = 18), with contig end sizes from 500-Kb to 2.5-Mb by the step of 500-Kb, and contact cutoffs from 1 to 5 times of the turning point by the step of 0.5 times, using both raw and normalized Hi-C matrix data from HiC-Pro. For smaller contig end sizes such as 500-Kb and 1.0-Mb, EndHiC tends to generate segmental results. For smaller contact cutoff such as 1 times of the turning point, EndHiC tends to over-scaffolding, which generates mis-joined results. By merging all the scaffolding results from various parameters, EndHiC can produce the correct chromosome-level scaffolds at most times.

**Table S3**. Contig lengths of the simulated data

1. Simulated contig assembly of Arabidopsis

| simulated contig id | size of contig (bp) | simulated contig id | size of contig (bp) |
| --- | --- | --- | --- |
| chr1_1 | 24,433,468 | chr1_2 | 5,994,203 |
| chr2_1 | 8,279,532 | chr2_2 | 11,418,757 |
| chr3 | 23,459,830 | chr4_1 | 3,274,824 |
| chr4_2 | 5,649,457 | chr4_3 | 9,660,775 |
| chr5_1 | 10,643,942 | chr5_2 | 7,618,754 |
| chr5_3 | 8,712,806 |  |  |

1. Simulated contig assembly of Rice

| simulated contig id | size of contig (bp) | simulated contig id | size of contig (bp) | simulated contig id | size of contig (bp) |
| --- | --- | --- | --- | --- | --- |
| chr1_1 | 4,668,004 | chr1_2 | 8,933,164 | chr1_3 | 10,594,129 |
| chr1_4 | 16,057,473 | chr1_5 | 3,245,294 | chr2_1 | 11,071,427 |
| chr2_2 | 5,591,207 | chr2_3 | 5,430,935 | chr2_4 | 13,670,634 |
| chr3_1 | 20,485,464 | chr3_2 | 7,403,842 | chr3_3 | 3,769,335 |
| chr3_4 | 5,358,678 | chr4_1 | 10,266,078 | chr4_2 | 14,041,159 |
| chr4_3 | 5,475,098 | chr4_4 | 5,877,967 | chr5_1 | 14,390,198 |
| chr5_2 | 10,388,512 | chr5_3 | 5,179,312 | chr6_1 | 9,428,046 |
| chr6_2 | 11,533,266 | chr6_3 | 3,730,237 | chr6_4 | 6,414,244 |
| chr7_1 | 16,607,388 | chr7_2 | 3,542,136 | chr7_3 | 9,331,154 |
| chr8_1 | 6,406,619 | chr8_2 | 7,178,526 | chr8_3 | 14,257,908 |
| chr9_1 | 4,047,212 | chr9_2 | 12,957,074 | chr9_3 | 5,936,576 |
| chr10_1 | 11,323,546 | chr10_2 | 5,000,429 | chr10_3 | 7,195,198 |
| chr11_1 | 9,453,280 | chr11_2 | 4,231,376 | chr11_3 | 15,088,244 |
| chr12_1 | 20,214,971 | chr12_2 | 3,391,462 | chr12_3 | 3,927,778 |

1. Simulated contig assembly of Human

| simulated contig id | size of contig (bp) | simulated contig id | size of contig (bp) | simulated contig id | size of contig (bp) |
| --- | --- | --- | --- | --- | --- |
| chr1_1 | 15,314,366 | chr1_2 | 62,862,049 | chr1_3 | 43,584,441 |
| chr1_4 | 26,466,065 | chr1_5 | 52,250,969 | chr1_6 | 47,909,438 |
| chr2_1 | 35,592,882 | chr2_2 | 4,548,005 | chr2_3 | 98,573,903 |
| chr2_4 | 10,732,293 | chr2_5 | 80,922,921 | chr2_6 | 12,326,748 |
| chr3_1 | 97,644,342 | chr3_2 | 29,851,964 | chr3_3 | 35,934,605 |
| chr3_4 | 17,551,867 | chr3_5 | 10,617,978 | chr3_6 | 9,505,192 |
| chr4_1 | 16,371,584 | chr4_2 | 80,714,469 | chr4_3 | 31,162,959 |
| chr4_4 | 13,566,375 | chr4_5 | 33,000,401 | chr4_6 | 18,759,157 |
| chr5_1 | 11,153,261 | chr5_2 | 48,698,539 | chr5_3 | 61,823,821 |
| chr5_4 | 20,753,805 | chr5_5 | 36,165,218 | chr5_6 | 3,450,795 |
| chr6_1 | 69,503,378 | chr6_2 | 18,306,112 | chr6_3 | 7,013,527 |
| chr6_4 | 28,271,167 | chr6_5 | 33,209,928 | chr6_6 | 15,822,516 |
| chr7_1 | 76,619,604 | chr7_2 | 13,931,025 | chr7_3 | 4,772,645 |
| chr7_4 | 24,195,672 | chr7_5 | 20,541,393 | chr7_6 | 20,507,089 |
| chr8_1 | 20,674,862 | chr8_2 | 8,354,705 | chr8_3 | 5,865,922 |
| chr8_4 | 86,849,161 | chr8_5 | 24,514,681 | chr9_1 | 10,364,324 |
| chr9_2 | 3,417,206 | chr9_3 | 12,080,080 | chr9_4 | 64,251,580 |
| chr9_5 | 20,716,544 | chr9_6 | 39,787,513 | chr10_1 | 24,909,837 |
| chr10_2 | 72,674,283 | chr10_3 | 9,371,747 | chr10_4 | 8,375,290 |
| chr10_5 | 19,426,977 | chr11_1 | 14,728,054 | chr11_2 | 34,951,681 |
| chr11_3 | 11,186,535 | chr11_4 | 18,952,781 | chr11_5 | 55,308,718 |
| chr12_1 | 6,824,554 | chr12_2 | 14,328,019 | chr12_3 | 9,415,298 |
| chr12_4 | 42,748,517 | chr12_5 | 60,008,160 | chr13_1 | 10,384,613 |
| chr13_2 | 29,425,364 | chr13_3 | 51,344,291 | chr13_4 | 14,532,355 |
| chr13_5 | 7,880,063 | chr14_1 | 21,802,899 | chr14_2 | 3,325,815 |
| chr14_3 | 3,974,784 | chr14_4 | 66,000,717 | chr14_5 | 6,057,277 |
| chr15_1 | 64,597,570 | chr15_2 | 4,378,550 | chr15_3 | 30,777,075 |
| chr16_1 | 16,056,673 | chr16_2 | 36,748,450 | chr16_3 | 43,525,251 |
| chr17_1 | 7,740,216 | chr17_2 | 66,415,412 | chr17_3 | 10,121,269 |
| chr18_1 | 13,204,932 | chr18_2 | 23,310,656 | chr18_3 | 44,026,950 |
| chr19_1 | 5,904,502 | chr19_2 | 55,802,862 | chr20_1 | 51,499,238 |
| chr20_2 | 14,711,017 | chr21_1 | 24,689,782 | chr21_2 | 20,400,900 |
| chr22_1 | 46,407,502 | chr22_2 | 4,917,424 | chrX_1 | 28,708,376 |
| chrX_2 | 27,740,406 | chrX_3 | 9,939,626 | chrX_4 | 10,905,426 |
| chrX_5 | 56,908,804 | chrX_6 | 20,056,928 |  |  |

**Table S4.** Summary of Hi-C data and HiC-Pro results

| **Species** | **Total bases of Hi-C data** | **Coverage depth (X)** | **Total read pairs** | **Uniquely aligned pairs** | **Self-circle** | **Dangling-end** | **Valid pairs** | **Valid pairs (duplicate removed)** | **Useful data percent** |
| --- | --- | --- | --- | --- | --- | --- | --- | --- | --- |
| Human | 124,972,344,900 | 41 | 416,574,483 | 124,673,886 | 33,998 | 2,938,444 | 110,014,494 | 98,391,593 | 23.6% |
| Rice | 21,470,420,100 | 58 | 71,568,067 | 45,777,278 | 34,487 | 79,982 | 45,002,791 | 40,821,917 | 57.0% |
| Arabidopsis | 10,288,604,600 | 86 | 102,886,046 | 45,244,994 | 978,212 | 381,050 | 37,791,223 | 21,220,262 | 20.6% |
| Great burdock | 201,613,850,100 | 117 | 672,046,167 | 269,305,617 | 134,310 | 93,807 | 268,112,029 | 212,922,075 | 31.7% |
| Water spinach | 64,406,145,600 | 133 | 214,687,152 | 102,797,519 | 74,093 | 48,041 | 101,439,749 | 73,091,154 | 34.0% |

Note: The human Hi-C reads data was downloaded from <https://github.com/marbl/CHM13>, while the Hi-C reads data for rice, Arabidopsis, great burdock, and water spinach were downloaded from NCBI-SRA (SRR5748737, SRR681003, PRJNA764011 and PRJNA764042). The statistics of uniquely aligned pairs, self-circle, dangling-end, valid pairs, and valid pairs (duplicate removed) were obtained from the result files of HiC-Pro. The restriction enzymes used in Hi-C library construction: Human (Arima), Rice (MboI), Arabidopsis (HindIII), Great burdock (MboI), and Water spinach (MboI).

**Table S5**. Usage instructions for the 6 compared Hi-C scaffolding tools

| Tool name | Usage example |
| --- | --- |
| Lachesis ( 21 Dec 2017) | # The input files are contig assemblies and bam files from HiC-Pro results.  # In the config file conf.ini, all the parameters were default parameters except CLUSTER_N, which should be determined by the chromosome number of a specific genome.  Lachesis conf.ini > Lachesis.out 2> Lachesis.err |
| AllHiC (v0.9.13) | # ALLHiC takes contigs and bam files from HiC-Pro results as inputs.  # For ENZYME: HindIII: AAGCTT; MboI: GATC; Arima: Arima. CHROMOSOME_NUMBER: the chromosome number of each plants.  ALLHiC_partition -b hicpro.botiew.bam -r draft.asm.fasta -e ENZYME -k CHROMOSOME_NUMBER -m 25  allhic optimize hicpro.botiew.counts_sites.*g1.txt hicpro.botiew.clm  ALLHiC_build draft.asm.fasta |
| 3D-DNA (v180922) | bwa index draft.fa  juicer/misc/generate_site_positions.py ENZYME draft draft.fa  juicer.sh -S early -g draft -s ENZYME -z ./references/draft.fa -y draft_ ENZYME.txt -p ./references/draft.fa.size -t 48 -D juicer/CPU  3d-dna/run-asm-pipeline.sh -r 0 -g 100 --sort-output draft.fa merged_nodups.txt |
| Pin_hic (v3.0.0) | bwa mem -SP -B10 -t20 ctgs.idx hic_R1.fastq.gz hic_R2.fastq.gz \| samtools view -b - > hic_mapped.bam  samtools faidx contigs.fa  pin_hic/bin/pin_hic_it -i 3 -x contigs.fa.fai -r contigs.fa hic_mapped.bam |
| YaHS (v1.2a.1) | # As suggested by YaHS, we used Arima Genomics' mapping pipeline (https://github.com/ArimaGenomics/mapping_pipeline) to preprocess HiC reads mapping.  sh mapping_pipeline.sh  # RESTRICTION_SITE: HindIII: AGCTT; MboI: GATC; Arima: GATC,GANT.  yahs --no-contig-ec -e RESTRICTION_SITE contigs.fa HiC_Arima_rep1.bam |
| HiC-pro (v2.11.4) & EndHiC (v1.0) | # HindIII: A^AGCTT; MboI: ^GATC; Arima: ^GATC G^ANTC;  HiC-Pro_2.11.4/bin/utils/digest_genome.py -r RESTRICTION_SITES -o contigs.bed contigs.fa  #Setting to proper paths in config file.  HiC-Pro_2.11.4/bin/HiC-Pro -i ./data/ -o ./hicpro_result -c ./config-hicpro.txt  #Run EndHiC with single round mode  perl endhic.pl Atha.contigs.fa.len AthaHiC_100000_abs.bed AthaHiC_100000.matrix AthaHiC_100000_iced.matrix  #Run EndHiC with multiple iterative rounds mode  perl endhic_iterate.pl --rounds 3 --binnumstep 5 contigs_all.len combine_100000_abs.bed combine_100000.matrix combine_100000_iced.matrix |

**Table S6**. Number of iterative rounds for the 6 Hi-C scaffolding tools

| **Scaffolding tools** | **Simulate datasets** | | | **Real datasets** | | |
| --- | --- | --- | --- | --- | --- | --- |
|  | Human | Rice | Arabidopsis | Human | Great burdock | Water spinach |
| **Lachesis** | 1 | 1 | 1 | 1 | 1 | 1 |
| **AllHiC** | 1 | 1 | 1 | 1 | 1 | 1 |
| **3D-DNA** | 17 | 11 | 5 | 164 | 14 | 48 |
| **Pin_hic** | 3 | 3 | 3 | 3 | 3 | 3 |
| **YaHS** | 10 | 8 | 8 | 10 | 9 | 8 |
| **EndHiC** | 1 | 1 | 1 | 2 | 1 | 1 |

Note: Lachesis and AllHiC used hierarchical agglomerative clustering algorithm, which do not have iterative scaffolding process, so the round numbers were recorded as 1; Pin_hic iterative model was used, and it iterated three times by default; 3D-DNA and YaHS determines the iterative round number automatically inside the tools.

**Table S7**.Statistics of simulated contigs used for mis-join detection

| Contig statistic | Arabidopsis | Rice | Human |
| --- | --- | --- | --- |
| Number of mis-joined contigs | 56 | 101 | 221 |
| N50 size of mis-joined contigs (bp) | 10,824,281 | 19,734,211 | 77,159,331 |
| N90 size of mis-joined contigs (bp) | 4,500,000 | 7,675,867 | 25,700,000 |
| Number of correct-joined contigs | 43 | 78 | 167 |
| N50 size of correct-joined contigs (bp) | 9,300,000 | 16,251,355 | 57,400,000 |
| N90 size of correct-joined contigs (bp) | 3,274,824 | 5,700,000 | 21,200,000 |

Table S8. Accuracy of EndHiC mis-join detection for simulated contigs

| Evaluation indicator | Simulated contigs from reference genome | | |
| --- | --- | --- | --- |
|  | Arabidopsis | Rice | Human |
| Contig number | 99 | 179 | 388 |
| True positives | 52 | 93 | 200 |
| False positives | 0 | 0 | 7 |
| True negatives | 43 | 78 | 160 |
| False negatives | 4 | 8 | 21 |
| Sensitivity | 0.93 | 0.92 | 0.90 |
| Specificity | 1.00 | 1.00 | 0.96 |

Note: Each chromosome of Arabidopsis, rice, and human was randomly split into 2 to 10 fragments, then any two fragments were randomly jointed to simulate mis-joined (erroneous) and correct-joined (error-free) contigs (size > 1 Mb). Then, comparable number of mis-joined and correct-joined contigs were chosen for the evaluation analysis. The corresponding Hi-C reads were mapped to the simulated contigs to generate contact matrixes using HiC-Pro pipeline, then EndHiC uses the *.bed and *.matrix files to detect mis-join errors in simulated contigs. True positives refer to the mis-joined contigs detected as mis-joined, False positives refer to the correct-joined detected as mis-joined, True negatives refer to the correct-joined contigs detected as correct-joined, and False negatives the mis-joined contigs detected as correct-joined, respectively. Sensitivity = True positives / (True positives + False negatives), Specificity = True negatives / (True negatives + False positives).

**Table S9.** Statistics of EndHiC scaffold assembly for 4 published plant genomes

| Species | EndHiC run | End size (Mb) | Contig/scaffold number | Total length (Mb) | N50 size (Mb) | Percent of genome assembly size (%) |
| --- | --- | --- | --- | --- | --- | --- |
| *Cichorium endivia* | input | - | 126 | 794.1 | 9.5 | 89.0 |
|  | round 1 | 0.5-2.5 | 32 | 768.1 | 29.7 | 86.1 |
|  | round 2 | 3.0-5.0 | 20 | 763.6 | 39.7 | 85.6 |
|  | manual | - | 9 | 763.6 | 87.4 | 85.6 |
| *Cichorium intybus* | input | - | 123 | 1244.2 | 15.9 | 97.3 |
|  | round 1 | 0.5-2.5 | 30 | 1230.1 | 59.6 | 96.2 |
|  | round 2 | 3.0-5.0 | 14 | 1225.2 | 117.9 | 95.8 |
|  | manual | - | 9 | 1225.2 | 144.7 | 95.8 |
| *Smallanthus sonchifolius* | input | - | 51 | 2669.3 | 87.4 | 98.3 |
|  | round 1 | 0.5-2.5 | 29 | 2654.2 | 97.0 | 97.7 |
| *Ipomoea cairica* | input | - | 20 | 726.3 | 43.8 | 99.1 |
|  | round 1 | 0.5-2.5 | 15 | 717.4 | 45.7 | 97.9 |

Note: genome assembly size is the total length of all the assembled contigs, but only the contigs with lengths > 1 Mb were used for the scaffold assembly by EndHiC.

**Table S10**. Statistics of EndHiC scaffold assembly with various contig lengths for human real data

| Total contig number | Average contig number | Total contig length (bp) | Contig N50 (bp) | Contig N90 (bp) | Round number | Complete chromo-some number | Mis-joined chromo-some number | Segment chromo-some number |
| --- | --- | --- | --- | --- | --- | --- | --- | --- |
| 80 | 3.48 | 3,054,815,472 | 70,781,613 | 15,830,159 | 1 | 23 | 0 | 0 |
| 104 | 4.52 | 3,054,815,472 | 47,909,438 | 14,532,355 | 1 | 23 | 0 | 0 |
| 131 | 5.70 | 3,054,815,472 | 34,322,354 | 11,861,935 | 2 | 23 | 0 | 0 |
| 148 | 6.43 | 3,054,815,472 | 33,723,829 | 9,152,058 | 2 | 23 | 0 | 0 |
| 197 | 8.57 | 3,054,815,472 | 20,685,232 | 7,771,459 | 3 | 23 | 0 | 0 |
| 404 | 17.57 | 3,054,815,472 | 13,075,384 | 3,912,578 | 3 | 21 | 0 | 2 |
| 628 | 27.30 | 3,054,815,472 | 9,415,532 | 2,453,269 | 3 | 14 | 0 | 9 |
| 860 | 37.39 | 3,054,815,472 | 6,729,763 | 1,853,337 | 3 | 8 | 0 | 15 |
| 1,773 | 77.09 | 3,054,815,472 | 3,128,780 | 942,192 | 3 | 3 | 0 | 20 |

Note: The human reference genome CHM13 v1.1 was used to simulate contig assemblies with various length level. When the contig N50 size is near or larger than 10 Mb, EndHiC can produce chromosome-level or near-chromosome-level scaffolds within 3 default rounds. For near-chromosome-level scaffolds, manual curation based on the Hi-C heatmaps can be adopted to get chromosome-level scaffolds.

**Table S11**. Performance comparison for preprocessing of the Hi-C scaffolding tools

| Preprocessing software | human | | rice | | Arabidopsis | |
| --- | --- | --- | --- | --- | --- | --- |
|  | **CPU time (m)** | **Peak Memory (Gb)** | **CPU time (m)** | **Peak Memory (Gb)** | **CPU time (m)** | **Peak Memory (Gb)** |
| HiC-Pro | 26,105.3 | 16.2 | 6,040.6 | 1.1 | 2,069.5 | 0.6 |
| Juicer | 20,003.9 | 186.4 | 4,474.0 | 94.6 | 801.4 | 25.2 |
| Bwa | 18,652.2 | 5.7 | 990.6 | 0.8 | 338.8 | 0.4 |
| Arima_map | 13,410.3 | 5.7 | 836.1 | 2.0 | 394.1 | 2.0 |

Note: The filtered bam files from HiC-Pro were used as input for Lachesis and AllHiC, the bin matrix files from HiC-Pro were used as input for EndHiC. The merged_nodups.txt file generated from Juicer pipeline was used as input for 3D-DNA. The resulting bam file from Bwa was used as input for Pin_hic. The resulting bam files from Arima_map pipeline was used as input for YaHS. In summary, the time consumption is: HiC-Pro > Juicer > Bwa & Arima_map.
